# Sex bias in tumor immunity: insights from immune cells

**DOI:** 10.7150/thno.106465

**Published:** 2025-03-31

**Authors:** Xuerui Tao, Yiling Wang, Binghua Xiang, Dongmei Hu, Wei Xiong, Wenjun Liao, Shichuan Zhang, Chi Liu, Xiaoxiao Wang, Yue Zhao

**Affiliations:** 1 School of Medicine, University of Electronic Science and Technology of China, Chengdu, China.; 2 Department of Radiation Oncology, Radiation Oncology Key Laboratory of Sichuan Province, Sichuan Clinical Research Center for Cancer, Sichuan Cancer Hospital & Institute, Sichuan Cancer Center, Affiliated Cancer Hospital of University of Electronic Science and Technology of China, Chengdu, China.; 3 Department of Urology, Sichuan Provincial People's Hospital, School of Medicine, University of Electronic Science and Technology of China, Chengdu, China.; 4 Department of Nephrology, Sichuan Provincial People's Hospital, School of Medicine, University of Electronic Science and Technology of China, Chengdu, China.; 5 Department of Organ Transplantation, Sichuan Provincial People's Hospital, School of Medicine, University of Electronic Science and Technology of China, Chengdu, China.

**Keywords:** Tumor immunity, Sex bias, Tumor microenvironment, Sex hormones, Immunotherapy

## Abstract

Significant sex disparities have been observed in cancer incidence, treatment response to immunotherapy, and susceptibility to adverse effects, affecting both reproductive and non-reproductive organ cancers. While lifestyle factors, carcinogenic exposure, and healthcare access contribute to these disparities, they do not fully explain the observed male-female variation in anti-tumor immunity. Despite the preferential expression of sex hormone receptors in immune cells, X chromosome also contains numerous genes involved in immune function, and its incomplete inactivation may enhance anti-tumor immune responses in females. In contrast, loss or downregulation of Y-linked genes in males has been associated with an increased cancer risk. Additionally, estrogen, progesterone and androgen signaling pathways influence both innate and adaptive immune responses, contributing to sex-specific outcomes in cancer progression and therapy. Sex-biased differences are also evident in the epigenetic regulation of gene expression, cellular senescence, microbiota composition, metabolism, and DNA damage response, all of which impact anti-tumor immunity and immunotherapy treatment efficacy. In general, the combination of sex chromosomes, sex hormones, and hormone receptors orchestrates the phenotype and function of various immune cells involved in tumor immunity. However, sex disparity in each specific immune cell are context and environment dependent, considering the preferential expression of hormone receptor in immune cell and sex hormone levels fluctuate significantly across different life stages. This review aims to outline the molecular, cellular, and epigenetic changes in T cells, B cells, NK cells, DCs, neutrophils, and macrophages driven by sex chromosomes and sex hormone signaling. These insights may inform the design of sex-specific targeted therapies and leading to more individualized cancer treatment strategies.

## 1. Background

Although men and women share nearly identical genomes, they exhibit distinct susceptibilities to cancer and differences in prognosis. The sex bias is observed not only in sex-specific cancers like breast and prostate cancer, but also in cancers originating from non-reproductive organs 1. In general, male patients experience higher mortality rates and shorter survival compared to females 2. While social behaviors, environmental exposures, and access to medical care contribute to these disparities, they account for only a fraction of the overall cancer risk 3. A prospective cohort study on 21 cancer types found that men have a higher risk of developing cancer at most shared anatomic sites compared to women. This increased risk persists even after accounting for behavioral risk factors and carcinogenic exposures, suggesting that sex-related biological factors including sex-biased immunity, may play a key role 3.

Sex-biased immunity has been well established both in innate and adaptive immunity, which have been observed in conditions such as inflammatory diseases 4, organ transplantation 5-7, systemic lupus erythematosus (SLE), various autoimmune disorders 8, as well as in COVID-19 9 and ischemic stroke 10. These differences also influence tumor immunity, which constantly participated in the process of oncogenesis, cancer progression, treatment responses and immune-related side effects. Factors contributing to cancer immune variability between genders include sex hormones, sex chromosomes 11, 12.

Tumor immunity can be affected by sex hormones, especially estrogen and androgen though its receptors in innate and adaptive immune cells. Estrogen signaling is primarily driven by the intracellular estrogen receptor (ER)-estrogen response element (ERE) complex 13, 14, which forms when ER bind to the ERE on DNA, either as homodimers or heterodimers 15, 16. The binding inevitably leads to the rapid activation of several signaling pathways, including the extracellular signal-regulated kinase (ERK)/mitogen-activated protein kinase (MAPK) pathway, cAMP-regulated gene transcription, protein kinase C (PKC)-mediated increases in intracellular Ca^2+^ levels, and JNK/phosphatidylinositol 3 kinase (PI3K) pathways, which regulate apoptosis 17. Classical membrane-bound 17β-estradiol (E2)-ER binding triggers multiple rapid signaling cascades 18, 19. Androgen signaling begins when the androgen receptor (AR) binds to its ligand, dihydrotestosterone (DHT), triggering a conformational change. This causes the AR to dissociate from heat shock proteins and enter the nucleus, where it undergoes phosphorylation and dimerization. The activated AR then binds to androgen response elements (AREs) in the promoters of target genes, recruiting co-activators to form transcriptional complexes. This process ultimately drives the transcription of genes that promote cancer progression and invasion 17, 20. However, sex hormones can ignite anti-tumor immunity and induce pro-tumor immunity at the same time through signaling on various immune cells, e.g., ER signaling pathways enhance the anti-tumor effects of most immune cells, while concurrently impairing macrophage-mediated tumor immunity in endometrial cancer, leading to the complexity of understanding the roles of each hormone.

Factors contributing to tumor immune responses include sex chromosomes and subsequent epigenetic modifications and coding genes in different immune cells as well. X-chromosomes are equipped with more immune functional genes including *TLR7*, *FOXP3*, and *CD40L*, and epigenetic modifying genes such as *KDM6A*, while X chromosome inactivation (XCI) upregulates or impairs anti-tumor immunity of various immune cells 21. Y chromosome genes, once thought to primarily regulate autoimmune diseases 22, may also impact cancer risk. Y chromosome mosaic loss (LOY) has been linked to an increase in regulatory T cell (Treg) numbers and activity, potentially heightening cancer susceptibility 23.

In general, both innate and adaptive immunity tend to be stronger in women, though the impact of sex hormone signaling and chromosomal differences on specific immune cells can be context dependent. For instance, tumor antigen presentation by dendritic cells (DCs) is enhanced in females through E2 and estrogen receptor alpha (ERα) signaling, which upregulates MHC class II expression and leads to more robust anti-tumor T cell responses. Additionally, macrophage polarization toward the M1 phenotype is promoted by AR signaling, which plays a role in immune response modulation 24, 25. In contrast, male T cells lack certain X-linked cytotoxic genes, and AR signaling in males promotes CD8^+^ T cell exhaustion, fostering immune tolerance within tumors. Estrogen has been shown to facilitate anti-tumor Th1 differentiation and encourage the differentiation of tolerogenic Tregs in CD4^+^ T cells 12. Of note, estrogen receptor beta (ERβ) signaling enhances CD8^+^ T cell activation and cytokine production through T cell receptor (TCR) stimulation, ultimately reducing tumor burden 26-28.

Improved understanding of sex biases in different immune cell may provide the insights for personalized patient management and treatment of cancer. In this context, we discuss the sex-biased molecular, cellular, and epigenetic regulation of anti-tumor immunity in various immune cells, shaped by a combination of hormonal and chromosomal factors.

## 2. Sex bias in T cell tumor immunity

T cells in the tumor microenvironment (TME) are heavily influenced by cancer-related factors. Previous research has focused on the anti-tumor mechanisms of CD4^+^ T cells and the two-step dysfunction of CD8^+^ T cells induced by the TME 29, 30. Recently, studies have identified the TME can induce a form of multi-reprogramming in T cells, which may contribute to their dysfunction. This finding offers new insights into cancer immunology and therapeutic strategies 30-38. However, there remains a lack of research on how the TME influences pro-T cell selection and maturation. Understanding these processes could provide valuable clinical insights and pave the way for new cancer immunotherapy.

Numerous studies have demonstrated that sex hormones influence T cell function, including T-cell development, gene expression regulating and epigenetic and metabolic reprogramming, through sex hormone receptors and their downstream signaling pathways 39-41. Specifically, progesterone (P4) and human chorionic gonadotropin (hCG) influence T cell epigenetic and metabolic reprogramming by upregulating progesterone-induced blocking factor (PIBF) and promoting histone methylation 42-44. This leads to an overall Th2 bias and an increased proportion of Tregs, which negatively impact tumor immunity 44-46. Recent research also identifies hCG as an autocrine tumor growth factor, and the expression of the progesterone receptor (PR) has been shown to impair the sensitivity of tumors to anti-LAG3 immunotherapy 47, 48.

**Figure 1:**
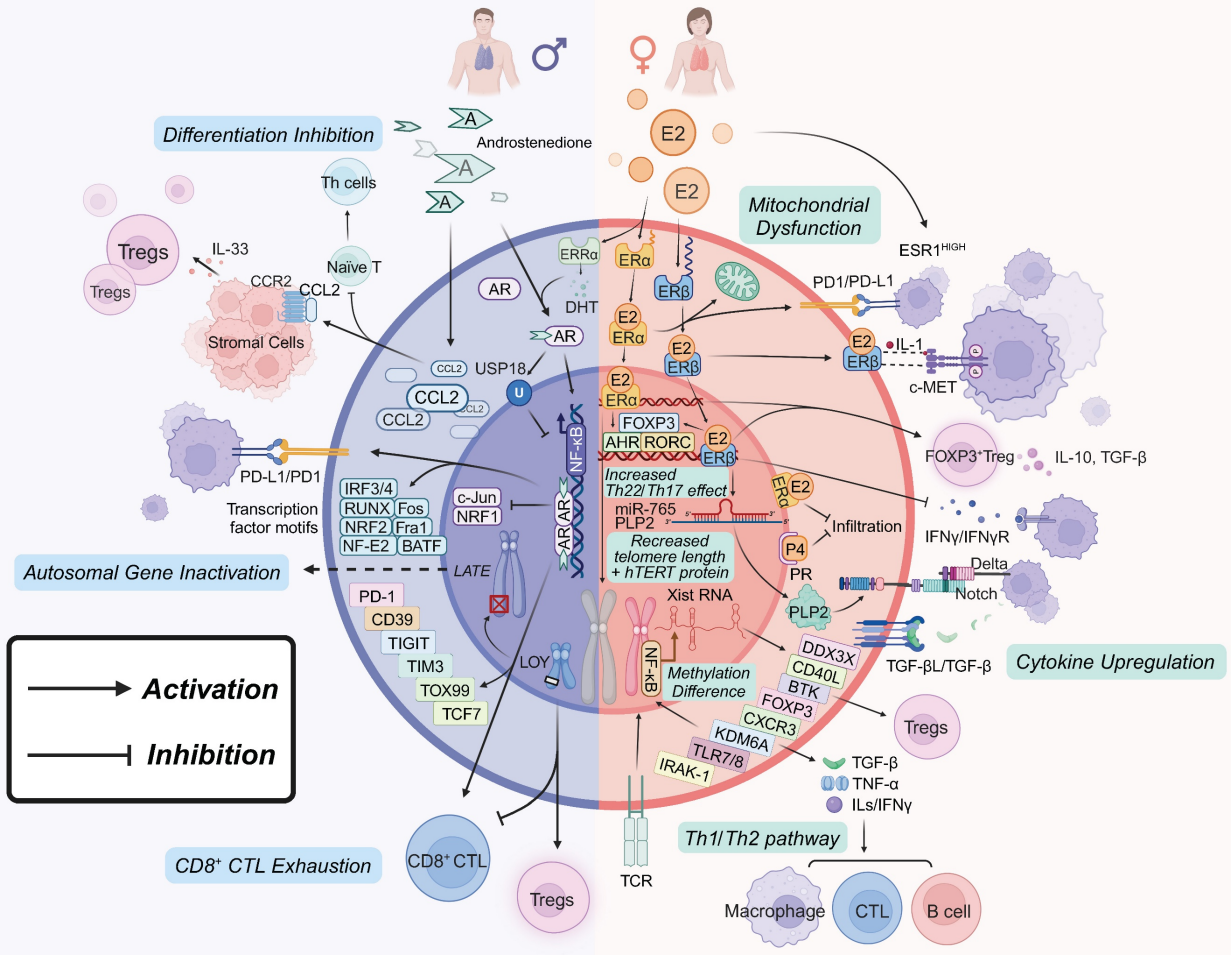
** Sex Factors in Regulating T Cell Tumor Immunity.** Estrogen effects: ERα Pathway: Promotes Th2 cell differentiation and proliferation by upregulating IL-4, IL-5, IL-6, IL-10, and IL-13, while concurrently suppressing Th1 responses. It enhances the proliferation of FOXP3^+^ Tregs and the expression of RORC, AHR, FOXP3, and PD-1, contributing to immune tolerance and promoting Th17/Th22 infiltration. High ERα expression is associated with reduced CTLs infiltration and mitochondrial dysfunction. ERβ Pathway: Modulates T cell function through the ERβ/c-MET and ERβ/IL-1/c-MET pathways, particularly in bladder cancer. The ERβ/miR-765/PLP2/Notch axis plays a significant role in the development of endometrial cancer and mediates immune escape in NSCLC. ERβ signaling also enhances Tregs differentiation and the secretion of IL-10 and TGF-β. Androgen effects: AR signaling suppresses T cell activation and differentiation by inducing USP18 and inhibiting NF-κB, leading to increased checkpoint molecule expression and subsequent CTLs exhaustion. In VAT, androgens may function independently of AR to promote IL-33 expression and Tregs recruitment, while also inhibiting T helper cells differentiation. Sex chromosomes and epigenetic effects: Evasion of XCI impacts T cell function by influencing genes involved in Th1/Th2 pathways and promoting FOXP3^+^ Tregs. LOY is associated with increased numbers and activity of Tregs, reduced CTLs exhaustion, and altered T cell gene expression, all of which influence cancer susceptibility. Sex hormones influence DNA methylation and histone modifications, affecting T cell functions, cancer progression and the enrichment of certain transcription factor motifs, while decreasing others, promoting Tregs' immunosuppressive capacity.

The concept of sex hormone regulated tumor responses is widely accepted, although the effects of androgens remain less well understood 49. Furthermore, there are significant sex-based differences in the differentiation of CD4^+^ and CD8^+^ T cells. Mechanisms such as X-chromosome gene linkage, Xist-mediated XCI evasion 50, Y-chromosome loss 23, and epigenetic inheritance 51 have all been implicated in modulating T cell function. Sex bias dependent T-cell exhaustion However, non-receptor-mediated pathways through which sex hormones affect T cells still require further exploration (Figure 1).

### 2.1 Estrogen regulation of T cells

Estrogen influences CD4^+^ T cells primarily through ERs in the TME, which enhance T helper (Th) 1 and Th2 immune responses and modulate cytokine and transcription factor secretion. Notably, E2 reduces the Th1/Th2 ratio, promotes Th22 and Th17 cell infiltration, and increases Th2-type cytokines, such as IL-4, IL-5, IL-6, IL-10, and IL-13, with IL-22 being particularly elevated 52. Elevated levels of E2 in the TME further enhance Th2 responses by downregulating IRF1 and increasing IL-4 production, thereby inhibiting Th1 activity and promoting Th2 differentiation and proliferation 53. E2 also enhances Th22 cell activity through the AHR/ARNT/NF-κB/ERK pathway and the RORC/OREs pathway, which upregulate AHR and RORC levels. Additionally, the *HER2* gene has been identified as a critical regulator of peripheral Th22 cell differentiation, working in tandem with Th17 cells to promote tumor growth 52.

Research into ERα and ERβ signaling in T-cell tumor immunity and targeted cancer therapy has provided valuable insights, but the full scope of the sex hormone signaling network remains incompletely understood. E2 promotes the proliferation and infiltration of FOXP3^+^ Tregs and induces the expression of programmed death-1 (PD-1) on their surface in an ER-dependent manner. This process is also thought to be mediated by GPER, which contributes to tumor immune tolerance 39, 54. ERα signaling enhances Tregs infiltration into the TME and upregulates the expression of immune-suppressive molecules like TGF-β and IL-10 by directly interacting with the FOXP3 promoter, thereby inhibiting effector T cell activation 55. Similar effects have been observed in non-small cell lung cancer (NSCLC), where increased ERα and PR gene expression correlates with reduced infiltration of CD4^+^ and CD8^+^ cytotoxic T lymphocytes (CTLs) 56, 57. ERα, as a regulatory binding protein (RBP), has also been shown to reduce telomere length, mitochondrial DNA copy number, and hTERT protein expression, thus inhibiting CTLs proliferation 58. Elevated *ESR1* gene expression has been linked to upregulation of immune checkpoint genes 59. Alternatively, ERα signaling may induce mitochondrial dysfunction in T cells 60. Additionally, estrogen can be converted to dihydrotestosterone (DHT) via ERRα, further activating downstream signaling pathways 61.

In contrast, ERβ is primarily expressed on immune cells rather than cancer cells, where it plays a complex and generally negative role in cancer immunology. It has been shown to decrease CD8^+^ CTLs infiltration and cytokine release, while promoting Tregs differentiation. In bladder cancer, CD4^+^ T cells facilitate tumor invasion through the ERβ/c-MET and ERβ/IL-1/c-MET pathways. Inhibition of IL-1 or the use of the ERβ antagonist PHTTP can reduce the malignancy of bladder cancer 62. Mutations in ERβ lead to increased infiltration of T cells and neutrophils in the TME 63, along with decreased interferon gamma (IFN-γ) levels, which promote tumor growth and progression 64. ERβ signaling in CD8^+^ T cells can activate these cells, enhancing the efficacy of αPD-1 tumor therapy by modulating tumor-derived phosphotyrosine 27. In prostate cancer, the estrogen/ERβ pathway can be inhibited to promote tumor invasion 65, 66. Moreover, miR-765 from CD45RO^-^CD8^+^ T cells can limit estrogen-driven endometrial cancer development through the ERβ/miR-765/PLP2/Notch axis 26. E2 can also impair cytotoxic of CTLs and facilitate immune escape in NSCLC cells via the ERβ/SIRT1/FOXO3a/PD-L1 axis 67. Furthermore, ERβ signaling promotes Tregs differentiation and induces their secretion of IL-10 and TGF-β, reducing CD8^+^ T cell cytotoxicity and overall tumor immunoreactivity 68, 69. Blocking ERβ-specific signaling can impair E2-mediated differentiation and function of intestinal Tregs, disrupting immune tolerance, as seen in female patients with chronic inflammatory bowel disease 70.

As the negative role of estrogen in various diseases becomes increasingly recognized, and with advancements in high-throughput technologies, platforms for discovering anti-estrogenic drugs have been developed. These platforms, combined with computational simulation techniques like molecular dynamics, have enabled the identification and efficacy prediction of estrogen and its receptor targets, as well as the development of novel therapeutic agents 71-77. For example, the use of the ERβ agonist S-oxynivalenol in combination with αPD-1 immunotherapy has shown promise in enhancing tumor-infiltrating CD8^+^ T cells. Moreover, improving the outcomes of immune checkpoint inhibitor therapy (ICT) may involve counteracting the potential downregulation of PD-1/PD-L1 expression by ERα through the IL-17 signaling axis 27, thus promoting CD8^+^ T cell infiltration 78. In summary, targeted therapies that address metabolic, epigenetic, and cancer-related pathways highlight the complex role of sex hormones in cancer progression and treatment 79.

### 2.2 Androgen regulation of T cells

Androgens play a crucial role in shaping T cell phenotypes, primarily through the AR signaling pathways. ARs in T cells are classified into intracellular androgen receptors (iAR) and membrane androgen receptors (mAR), both of which regulate T cell development, differentiation, and immune response 80-82. The iARs, also known as cytosolic ARs, interact with key signaling pathways, including PI3K, Src family kinases, RAS GTPases, and the MAPK/ERK pathway, where it regulates cell proliferation and survival. In contrast, the non-classical mARs interact with ERK1/2 pathways and G proteins, showing a different androgen affinity compared to iARs 83. However, distinct androgen receptor signaling functions have not yet been identified in T cells or other immune cells. In detail, androgen and AR signaling can inhibit NF-κB activation, which in turn suppresses T-cell development, maturation, Th1 differentiation, and proliferation 84-86. Additionally, androgens can modulate the expansion and function of Tregs by increasing the expression of FOXP3 87. These processes impair tumor immunity and help prevent autoimmune diseases in males by promoting immune tolerance through an AREs-dependent mechanism 84, 88.

Recent advancements in tumor immunotherapy, including androgen deprivation therapy (ADT), have rekindled interest in the effects of androgens and AR signaling on T cell-mediated tumor immunity. ADT, commonly used in prostate cancer treatment, has been recognized for its ability to block AR signaling in T cells, thereby restoring T cell activation by inhibiting the PD-1/PD-L1 pathway 81. This therapeutic effect suppresses Th differentiation in naïve T cells while promoting Tregs activation 64. Androgen signaling has also been shown to contribute to cancer-related immunosuppression by suppressing T cell activity through the upregulation of USP18, which inhibits NF-κB activity 84. Additionally, AR activity in T cells can lead to reduced IFN-γ production and resistance to tumor immunotherapy, diminishing the effectiveness of immune checkpoint inhibitors (ICIs). This downregulation may be counteracted by combining AR antagonists with PD-1 inhibitors 89.

ER signaling is integral to this process collectively. For example, aromatase gene knockout (KO) mice, which are unable to produce estrogen, do not develop prostate cancer despite elevated testosterone levels 90. Recent studies have shown that estrogen promotes endoplasmic reticulum-associated degradation and enhances the expression of the proto-oncogene c-Myc in prostate cancer cells through AR/ER signaling, highlighting the complex mechanisms underlying sex hormone action 91. In contrast, in visceral adipose tissue (VAT), androgens may act independently of AR in a sex-hormone-dependent manner to stimulate IL-33 expression in stromal cells via the CCL2/CCR2 axis 49, 92. This mechanism increases Tregs recruitment and results in higher Tregs levels in males, thereby contributing to more effective restriction of VAT inflammation.

T-cell exhaustion progression, a factor that directly influences cancer progression and therapeutic response, is significantly modulated by androgens. It progresses more rapidly in male T cells than in female T cells, with males exhibiting a higher frequency of progenitor exhausted T cells (PEX; CD8^+^CD44^+^PD-1^+^TCF1^+^TIM3^-^). However, sex bias in terminally exhausted T cell (TEX; CD8^+^CD44^+^PD-1^+^TCF1^-^TIM3^+^) remains to be illustrated 93, 94. In contrast, female tumor-infiltrating T cells show elevated frequencies of effector T cells (EFF; CD8^+^CD44^+^TCF1^-^TIM3^-^), along with a mild increase in inhibitory receptor expression and a decrease in cytokine expression 94. The availability and expression of AR in intratumoral stem cell-like CD8^+^ T cells may drive differentiation toward terminal exhaustion and a PEX signature, impairing endogenous T cell stemness and function within the TME 95, 96. Therapeutic CD8^+^ T cells with AR deficiency or blockade demonstrated a reduction in the terminally exhausted subset, downregulated immune checkpoint expression, and enhanced cytotoxicity and tumor immunity 89, 95. Recent research has revealed that AR signaling plays a key role as a T cell-intrinsic regulator of CD8^+^ T cell exhaustion and in regulating the differentiation of stem cell-like CD8^+^ T cells into a terminally exhausted phenotype through transcriptional mechanisms 96, 97. This is evidenced by the overexpression of T-cell immune checkpoints such as PD-1, CD39, TIGIT, LAG3, CTLA4 and TIM-3 infiltrating in tumors compared with orchiectomy or female group 84, 94. Anti-PD-1 blockade is also more effective in male T cells, resulting in a significant reduction of exhausted T-cell subsets and increased proliferation compared to females. These changes may contribute to a survival benefit in males, as reported in studies 94. The underlying mechanism may involve AR acting as a trans-activator of T cell factor (TCF) 7 and a sex-specific TCF7-centered regulon in CD8^+^ T cells 96, 98. Additionally, CUT&Tag-seq analyses have shown that AR directly binds to the promoters of key transcriptional regulators of T cell exhaustion, including TCF7 and TOX 97, 99.

AR signaling also mediates epigenetic changes in T cells, resulting in increased enrichment of transcription factor motifs such as Fra1, Fos, NF-E2, RUNX, IRF3, IRF4, NRF2 (NFE2L2), and BATF, while decreasing the enrichment of c-Jun and NRF1 motifs in male 2C-TCR CD8^+^ T cells in antitumor immunity 97. Meanwhile, AR pathways induced epigenetic changes can lead to metabolic and epigenetic reprogramming of T cells 100. Androgens inhibit the expression of Delta-like 4 (Dll4), a Notch ligand essential for T cell progenitor commitment and differentiation, in a dose-dependent manner in cortical thymic epithelial cells (cTECs) 101. This regulation occurs through androgen-response elements in the *Dll4* gene promoter, where androgen/AR complexes are recruited, as demonstrated by chromatin immunoprecipitation 101. In non-TME contexts, androgens promote self-tolerance by upregulating the expression of autoimmune regulator (Aire) in medullary thymic epithelial cells (mTECs). This occurs through direct binding to the Aire gene promoter, resulting in more efficient negative selection of self-reactive T cells 102, while estrogen decrease the expression of Aire by epigenetically regulating its methylation 103.

### 2.3 Sex chromosome regulation of T cells

Sex differences in T cell phenotypes can be attributed to Xist-mediated XCI evasion 50. Key signaling pathways, such as TCR and NF-κB, play a role in maintaining these differences. Several genes that evade XCI in CD4^+^ and CD8^+^ T cells, including *CD40L*,* CXCR3*, *IRAK-1*, *BTK*, *DDX3X*, and *TLR7/TLR8*, influence Th1 and Th17 differentiation and are linked to autoimmune conditions 104. These genes are more prevalent in females 50, 105, 106. For example, the *FOXP3* gene, which is crucial for tumor resident Tregs function, is more highly expressed in female CD8^+^ T cells and tumor resident T cells due to XCI evasion, contributing to decreased tumor immunoreactivity 107, 108. The *KDM6A* gene, which escapes XCI and produces UTX, modulates Th1 and Th2 pathways and influences immune function in NK cells, leading to more pronounced autoimmune responses in females 39, 109. *KDM6A* has also been identified as a critical target for immunotherapy in glioblastoma. In male glioblastoma patients, lower *KDM6A* expression compared to females is linked to higher levels of immune suppression and T-cell exhaustion, resulting in a less effective immunotherapy response 110. The reduction in T-cell durability, influenced by UTX in the chronic inflammatory cancer microenvironment, may explain this difference 111.

Although research on the X chromosome's role in cancer remains limited, a recent review highlights the similarities in T cell behavior, including epigenetic modifications, gene expression, cytotoxicity, and especially exhaustion, in chronic inflammation and autoimmune diseases 112, 113. Many studies have integrated the role of T cells in the three conditions to explore these areas collectively 112, 114, 115. A clinical review also suggested that autoimmune responses may be beneficial for cancer prognosis 116. We anticipate that the influence of sex chromosome-related factors on tumor-infiltrating T cells will be uncovered in future research, and these findings could contribute to the development of X chromosome-targeted therapies for cancer.

Recent findings also suggest that Y chromosome genes, once thought to primarily regulate autoimmune diseases 22, may also impact cancer risk. Y chromosome mosaic loss (LOY) has been linked to an increase in Tregs numbers and activity, potentially heightening cancer susceptibility 23. T cells with LOY exhibit altered expression of autosomal genes, such as *LATE* 117, which can contribute to CD8^+^ T cell exhaustion and dysfunction, facilitating tumor escape from T cell-mediated killing 118. Interestingly, LOY tumors have shown increased sensitivity to ICI therapy, suggesting a potential avenue for effective treatment 118.

### 2.4 Epigenetic regulation of T cells

Epigenetic changes governed by sex hormones and chromosomes offer promise for future treatments of "cold" tumors, particularly when combined with ICIs and adoptive T cell therapy 119. The sex-differentiated manifestations observed in various allergic diseases and asthma are often regulated by methylation and histone modifications of relevant genes. For instance, genes such as *IFNG*, *IL17A*, and *IL17F* in Th cells, as well as *RORC*, *FOXP3*, and *IL4* in Tregs, display high levels of H3K9me2 marking, while methylation markers in different T cell subsets exhibit considerable variation 120. This mechanism may stem from multiple ERα-binding sites within the promoters of DNA methyltransferase isoforms DNMT1, DNMT3A, and DNMT3B, along with the sex-specific effects of the dosage compensation mechanism that inactivates the female X chromosome, impacting methylation sites on the X chromosome 121.

Moreover, epigenetic mechanisms involved in carcinogenesis within T cells have been identified. For example, the normalization of FOXP3 expression relative to Tregs numbers affects the non-oxidative pentose phosphate pathway (PPP), leading to the downregulation of Tregs' immunosuppressive capacity due to a deficiency in transketolase (TKT), an essential enzyme in the non-oxidative PPP. This epigenetic downregulation increases the risk of carcinogenesis 122. The senescence characteristic of CD8^+^ T_EMRA_ cells in human blood is also influenced by the methylation of Cytosine-phosphate-Guanine dinucleotide (CpG) motifs 123, which could open avenues for therapeutic intervention.

Recent research has introduced methods such as EPRIM 124, which evaluates the pre-efficacy of anti-ER therapy based on epigenetic modifications, and WASp, a potential drug targeting epigenetic pathways to prevent transcription-associated DNA damage in malignant tumors by addressing epigenetic deregulation, such as clearing the R-loop. These approaches have been shown to promote the differentiation of Th cells in T-cell acute lymphoblastic leukemia (T-ALL) patients and inhibit T-ALL progression 125. However, further research is needed to fully understand the complex interactions between cancer development mechanisms, sex-specific factors, and T cell epigenetics, which may pave the way for novel therapeutic strategies.

## 3. Sex bias in B cell tumor immunity

**Figure 2:**
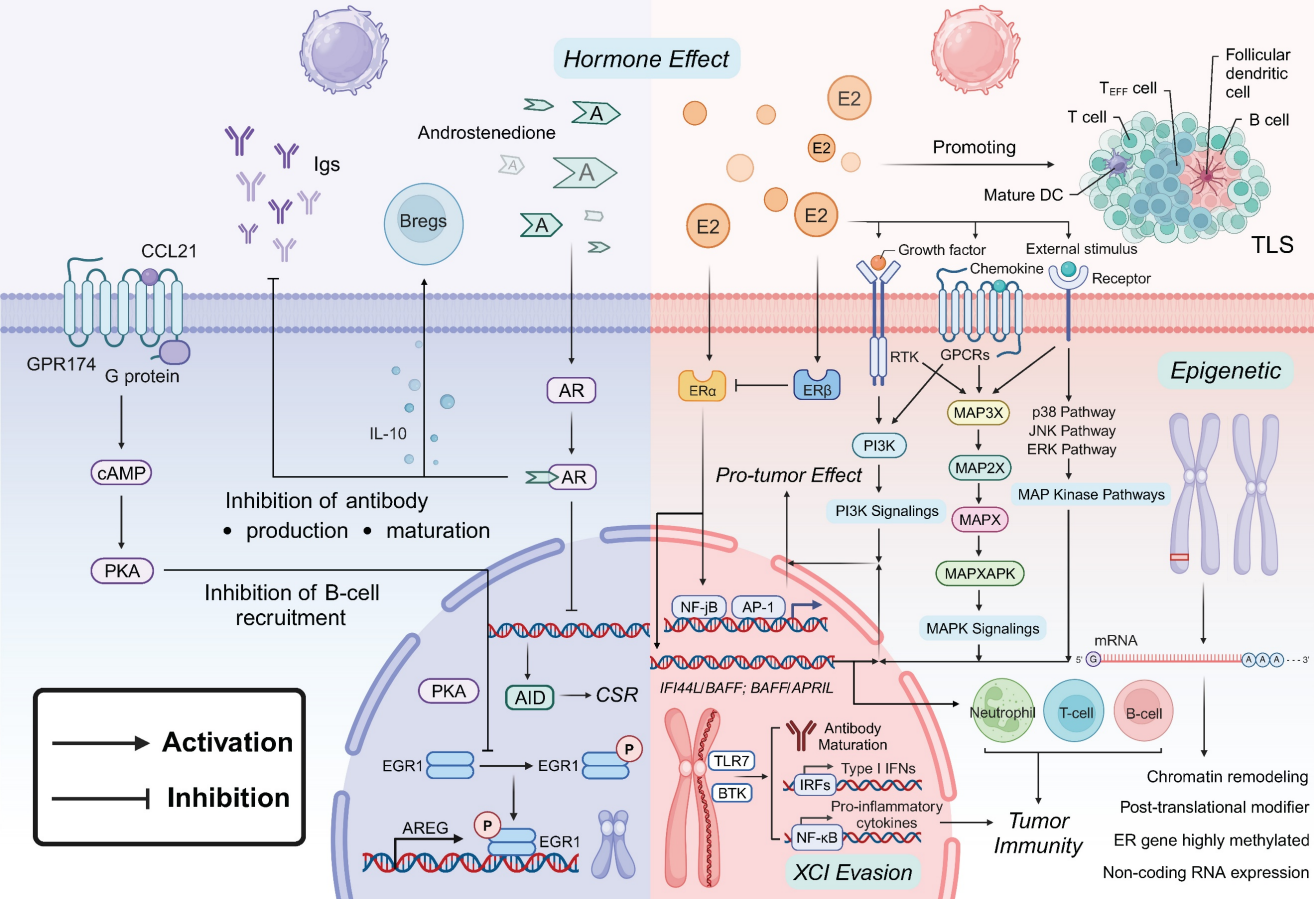
** Sex Factors in Regulating B Cell Tumor Immunity.** Hormone Effects on B Cells: Estrogen interacts with its respective receptors—ERα and ERβ, to promote B cell activation, antibody production, and maturation within the TME. Estrogen enhances growth factor receptor signaling and activates downstream PI3K and MAPK pathways, which contribute to pro-tumor effects, including the promotion of TLS. In males, androgens such as androstenedione act through AR to suppress B cell recruitment, inhibit antibody production, and reduce antibody maturation. The AR pathway negatively regulates B cell function by enhancing IL-10 production in Bregs, which in turn suppresses B cell activation and overall immune responses. Additionally, GPR174 signaling, modulated by the CCL21/GPR174 axis, inhibits B cell aggregation and function within germinal centers, further contributing to the suppression of antibody production and immune responses. XCI Evasion and Epigenetic Pathways: In females, the evasion of XCI results in the overexpression of genes such as *TLR7* and *BTK*, which are crucial for B cell proliferation and activation. The escape of *TLR7* from XCI, when combined with estrogen signaling, upregulates the IFI44L/BAFF pathway, thereby promoting IFN-I responses, enhancing immunoglobulin maturation and systemic autoimmune diseases and contributing to heightened inflammatory activation. Sex hormones drive significant epigenetic modifications in B cells, including ER gene methylation, post-translational modifier, non-coding RNA expression and chromatin remodeling. These changes influence gene expression, impacting tumor immunity and potentially leading to developing anti-estrogen resistance in cancer, translational repression of mRNAs, endocrine resistance, and drug resistance.

In disease models of SLE in females and males, including the Klinefelter syndrome model, B cell function is modulated by a range of molecular mechanisms, including sex hormones, sex chromosomes, and epigenetic factors 50, 126-128. These differences indicate that B cell-mediated immune responses in the TME may exhibit significant sex disparities (Figure 2).

### 3.1 Sex bias in TLS

Tumor-infiltrating B lymphocytes (TIL-Bs), comprising both tumor-infiltrating B cells and plasma cells, play a critical and multifaceted role in tumor immunity and are associated with improved clinical outcomes. These B cells contribute to the formation of tertiary lymphoid structures (TLS), which are aggregates of immune cells resembling lymph nodes and serve to establish localized and sustained tumor immune responses 129. TIL-Bs inhibit tumor progression through multiple mechanisms, including the secretion of immunoglobulins, which promote T cell responses, as well as cross-presenting antigens to T cells to either activate or directly kill them 129-131. Within TLS, TIL-Bs may function as antigen-presenting or regulatory cells, thereby influencing T cell activity 129. They are also involved in the formation, maturation, and maintenance of TLS, which has been shown to reduce tumor growth 132. The presence of high concentrations of B cells, particularly in TLS, is associated with better prognosis and improved responses to ICIs 133, 134, likely due to the TLS-induced infiltration of CTLs into the tumor 135, 136. Clinical studies indicate that the extent of TIL-B infiltration, particularly in breast cancer, is positively correlated with favorable outcomes. This highlights the potential of inducing tumor immunoreactivity in TIL-Bs through modulation of other immune cell subpopulations as a promising strategy for immunotherapy 137, 138.

Sex differences in B-cell-mediated tumor phenotypes are frequently observed in TMEs characterized by TLS versus B-cell tumors. These differences are believed to be mediated by sex hormone pathways, XCI, gene expression regulation, and epigenetic mechanisms. In patients with papillary thyroid carcinoma (PTC) and NSCLC, a higher area ratio of immune cell aggregates is observed in females, leading to more effective tumor immune surveillance and enhanced anti-tumor immunity 139. This may be attributed to the presence of more mature and diversified TLS in females, facilitated by sex hormone pathways. In pancreatic adenocarcinoma (PAAD), a significant positive correlation has been observed between the levels of estrogen receptors ESR1 and ESR2 and TLS scores in the TME. Female patients with PAAD show higher TLS scores and a better prognosis, suggesting that estrogens may play a pivotal role in promoting TLS formation and enhancing anti-tumor immunity 140. These TLS play a crucial role in the regulation of immune cell function and in supporting tumor antigen-driven activity within the TME 139.

### 3.2 Estrogen regulation of B cell malignancy

In B-cell lymphoma, female patients generally report a higher quality of life, experiencing reduced fatigue, pain, and anxiety/depression compared to their male counterparts 141. However, in diffuse large B-cell lymphoma (DLBCL), prognosis and survival rates decline significantly after menopause, suggesting that estrogens may have a regulatory effect on B-cell tumors 142. Estrogen's role in B-cell malignancies likely involves activation of downstream signaling pathways such as MAPK, PI3K, and the MAP kinase family. Estrogen has been shown to increase B-cell activating factor (BAFF) transcript levels in B cells, suggesting that estrogen-induced upregulation of IFI44L/BAFF or BAFF/APRIL expression may activate B cells, T cells, and neutrophils, contributing to immune modulation 143-146. Chimeric antigen receptor T-cell (CAR-T) therapies targeting B-cell cancers based on BAFF ligands have shown notable success 147. However, the BAFF/APRIL system can also act as an inhibitor of tumor infiltrating CD8^+^ T cells, inhibited by regulatory B cells (Bregs), and a pro-oncogenic factor in some tumors, complicating its role in tumor progression 145, 148.

Additionally, ER signaling regulates gene transcription through interactions with transcription factors like AP-1 and NF-jB 149. ERα-related signaling pathways promote cell proliferation and tumorigenesis, while ERβ typically acts antagonistically 150. Agonists and estrogen analogues that activate ERβ can inhibit angiogenesis and cancer cell proliferation, suppressing cancer development, except in chronic lymphocytic leukemia (CLL) 151, 152. ERβ-targeted antagonists, such as tamoxifen, have been shown to significantly induce cell death in DLBCL and reduce the risk of DLBCL in patients with ERα^+^ breast cancer, highlighting the therapeutic potential of targeting ERβ in B-cell lymphoma treatment 142.

### 3.3 Androgen regulation of B cell malignancy

Statistical analyses of DLBCL patients indicate that male gender is a negative prognostic factor for overall survival (OS) and progression-free survival (PFS). Men typically exhibit poorer prognosis and higher recurrence rates, likely due to the negative effects induced by androgens 153. In animal models, such as androgen receptor knockout (ARKO) mice, DHT has been shown ineffective in restoring B cell function. These mechanisms may involve androgen receptor signaling promoting gastric cancer (GC) cell genesis and progression through overexpression of miR-125b and inhibition of apoptosis 154. Similar mechanisms have been identified in bladder cancer as well 155. Furthermore, B cells in testicular feminization (Tfm) mice do not respond to androgens in bone marrow-derived cells (BMDCs) 156, indicating that androgen regulation of B cell function occurs via AR and possibly alternative pathways. One such pathway involves the X chromosome-encoded G protein-coupled receptor-174 (GPR-174), which is involved in the CCL21/GPR signaling axis. This signaling pathway inhibits B cell aggregation in the germinal centers of male mice, further emphasizing the complex role of androgens in modulating immune responses and cancer progression 157. In ER^+^ breast cancer, androgens inhibit ER-dependent cell proliferation while simultaneously upregulating BCL2 family proteins. This dual action has been associated with a more favorable prognosis in anticancer treatments, suggesting that androgen signaling might modulate the balance between pro-survival and pro-apoptotic factors in certain cancers 158.

### 3.4 Sex chromosome and epigenetic regulation of B cell malignancy

In nearly 90% of patients with human B-cell leukemia and lymphoma, as well as other significant tumors, high methylation of ER genes and low ER expression, along with noncoding RNA expression, chromatin remodeling, and posttranslational modifications, are observed 159-162. These factors contribute to translational repression of mRNAs, leading to endocrine resistance and drug resistance. Therapeutic strategies involving histone acetyltransferases (such as MYST3), deacetylases (like HDAC1), and demethylases (e.g., LSD1 and miR-29b) may help restore sensitivity to estrogens and ER expression, offering a potential avenue for overcoming resistance and improving treatment outcomes 162. In patients with DLBCL undergoing rituximab immunotherapy combined with chemotherapy, PFS significantly improves in women 163, which may be linked to sex differences in adipose tissue distribution 164.

## 4. Sex bias in NK cell tumor immunity

### 4.1 Estrogen regulation of NK cells

**Figure 3:**
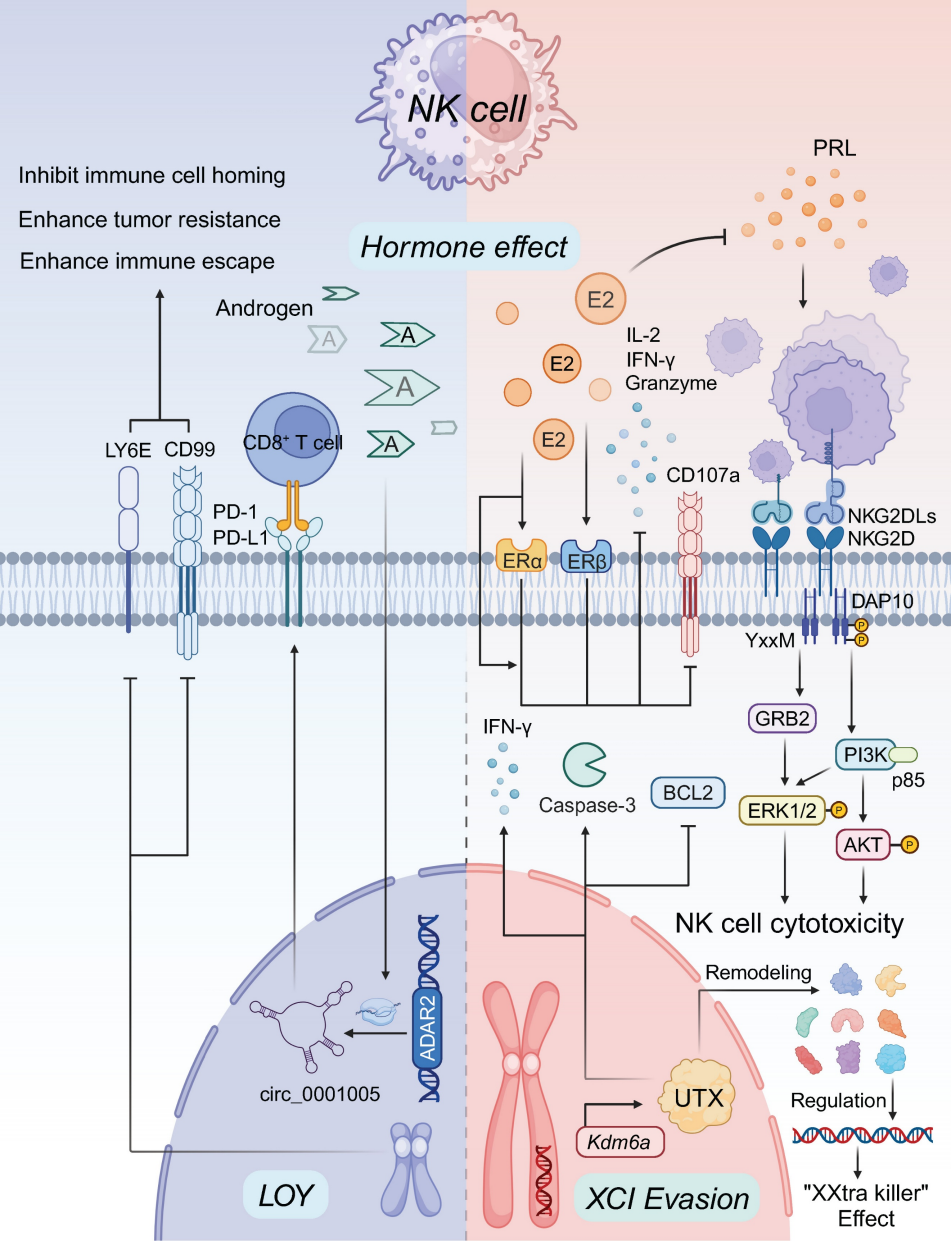
** Sex Factors in Regulating NK Cell Tumor Immunity.** E2 impacts on NK cell functions through its estrogen receptors, ERα and ERβ, including proliferation, cytotoxicity, granzyme B secretion, and the synthesis of IL-2 and IFN-γ. E2 also influences the expression of surface molecules like CD107a through both ER-dependent and non-ER-dependent pathways, depending on NK cell subtypes and context, with estrogen sometimes enhancing the immune response of NKT cells. PRL, has been observed to promote NK cell-mediated cytotoxicity in cervical cancer via the NKG2D/NKG2DL axis, counteracting some of the inhibitory effects of E2 on NK cell function. Androgen signaling is known to suppress NK cell immune responses, potentially by upregulating PD-L1. In models of antiandrogen treatment and AR knockout, downregulation of the ADAR2 gene product circRNA circ_0001005 in NK cells led to reduced PD-L1 expression, which indirectly enhanced NK cell-mediated tumor killing through increased CD8+ T cell activity. The KDM6A gene, evades XCI, leading to elevated UTX levels. UTX plays a crucial role in preventing NK cell quiescence and enhancing effector functions by remodeling chromatin and regulating gene expression. UTX-deficient NK cells show increased expression of the anti-apoptotic factor BCL2 and decreased IFN-γ production, leading to reduced NK effector function. LOY in NK cells is associated with downregulation of key immune homing molecules like CD99 and LY6E, which can impair NK cell-mediated immune surveillance and promote tumor immune escape.

The influence of estrogen on NK cells is complex and multifaceted, impacting various aspects of their function. Estrogen regulates NK cell proliferation, cytotoxicity, secretion of granzyme B, production of cytokines such as IL-2 and IFN-γ, and the expression of surface molecules like CD107a 64, 165-167. These effects occur through both ER-dependent and ER-independent mechanisms. NK cells express both ERα and ERβ on their surface, with the latter typically recognized as the negative target of estrogen's effects through a non-ERα-mediated pathway 168. However, recent research suggests that estrogen's impact on NK cell in peripheral blood activity may vary depending on the specific NK cell subtype 169. For example, estrogen has been shown to enhance the immune response of NKT cells 39. Estrogen deprivation therapies, such as selective estrogen receptor modulators (SERMs), have demonstrated promise in cancer treatment by stimulating NK cell and CD8^+^ T cell activation, particularly in peripheral blood from triple-negative breast cancer (TNBC) patients 170. In contrast, prolactin (PRL) has been found to enhance NK cell-mediated cytotoxicity in cervical cancer through the NKG2D/NKG2DL axis, which may counterbalance the effects of estrogen 171. While prolactin receptor signaling on NK cells is recognized, its influence is more prominent in conditions like recurrent miscarriages rather than in cancer-related contexts 172 (Figure 3).

### 4.2 Androgen regulation of NK cells

In contrast, androgen signaling is known to suppress NK cell immune responses. The blockade of AR has gained attention as a potential strategy for cancer therapy. In a study involving antiandrogen treatment and ARKO models, Qing Liu et al. discovered that the circRNA circ_0001005, encoded by the *ADAR2* gene, was downregulated in NK cells, leading to reduced PD-L1 expression. This decrease in PD-L1 facilitated NK cell-mediated tumor elimination by enhancing CD8^+^ T cell activity 173, 174. The inhibitory effect of androgens on NK cell cytotoxicity may involve upregulation of PD-L1 or suppression of IL-12A expression in tumor cells. Evidence suggests that androgen receptor antagonists improve tumor control in mouse models of bladder cancer 175-177. However, further research is needed to fully understand the direct impact of androgens on NK cell function.

### 4.3 Sex chromosome and epigenetic regulation of NK cells

Sex differences in NK cell activity are influenced not only by sex hormones but also by sex chromosomes and epigenetic factors. Studies have shown that female NK cells persistently secrete higher levels of IFNγ, perforin, and granzyme B compared to male NK cells in gonadectomized mice, suggesting that X-linked epigenetic regulators contribute to NK cell sex bias 178. Recent research has shifted focus from gonadotropins to the X-linked epigenetic regulator, ubiquitously transcribed tetratricopeptide repeat on chromosome X (UTX), which is encoded by the *KDM6A* gene 179. The *KDM6A* gene evades XCI, leading to elevated UTX protein levels. UTX regulates gene expression by remodeling chromatin, thus preventing NK cell quiescence and enhancing their effector functions, effectively making female NK cells "XXtra killer cells" 180, 181. In contrast, UTX-deficient NK cells show increased expression of the anti-apoptotic factor BCL2 and decreased IFNγ production, resulting in a higher frequency of NK cells but impaired effector function 50. Additionally, both male and female Utx^NKD^ cells exhibit altered caspase-3 activity and increased BCL2 expression, suggesting that UTX plays a critical role in NK cell homeostasis by promoting apoptosis 179. LOY has been linked to increased tumor incidence, aggression, and metastasis, potentially due to the downregulation of *CD99* and *LY6E* in NK cells 118, 182. This downregulation may impair immune cell homing, thereby enhancing tumor resistance and facilitating immune escape 183. Additionally, epigenetic factors, including DNA methylation, histone modifications, transcription factors, and miRNA regulation, play significant roles in modulating NK cell-mediated cytotoxicity 184. However, the precise functions of these factors, particularly in relation to sex-based differences, remain to be fully elucidated and warrant further investigation.

## 5. Sex bias in dendritic cell tumor immunity

**Figure 4:**
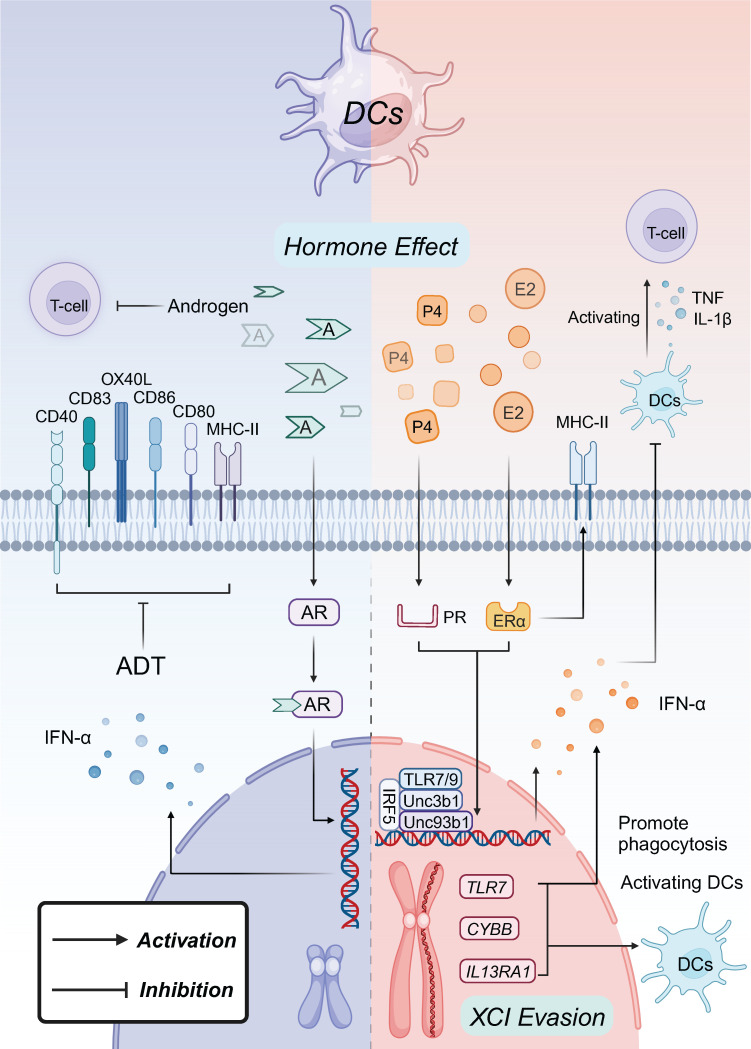
** Sex Factors in Regulating DCs Tumor Immunity.** Higher concentrations of P4 regulate DC phenotypes through interactions with PR signaling pathways, associated with the upregulation of TLR7, Unc93b1, and TLR9 in DCs, which enhances their activation and phagocytic abilities. P4 also correlates with increased levels of IFN-α^+^ pDCs in peripheral blood, which can inhibit TNF and IL-1β secretion by conventional DCs and downregulate their ability to activate T cells. E2 exerts similar effects by increasing the proportion of IFN-α^+^ DCs through upregulation of Unc93b1 and IRF5 expression via ERα dependent pathways. E2/ERα signaling enhances DC activation by upregulating MHC class II molecules and co-stimulatory molecules, thereby boosting their APCs efficacy. This signaling also increases environmental GM-CSF and IRF levels, further augmenting DC-mediated immune responses. The enhanced TLR7 and IFN signaling in female DCs, due to XCI evasion, contributes to the heightened production of IFN-α, particularly in pDCs. AR-mediated signaling exerts inhibitory effects on DC function, particularly in T cell activation and pDC responses. Androgens suppress the surface expression of MHC-II and co-stimulatory molecules (e.g., CD80, CD83, CD86, CD40, OX40L) on DCs, leading to reduced capacity to stimulate T cells. High androgen levels in male infants and adolescents are associated with lower IFN-α production by pDCs, and testosterone administration in transgender males also diminishes pDC production of IFN-I, further emphasizing the suppressive role of androgens on DC-mediated immunity. XCI evasion results in the overexpression of X-linked genes such as *TLR7*, *CYBB*, and *IL13RA1* in DCs, enhancing DC activation, phagocytosis, and IFN-α production, contributing to a more robust immune response compared to males.

While research on sex bias in dendritic cells (DCs) within the TME is limited, sex-specific disparities in DCs are still largely influenced by sex hormones pathways and other related factors, which may provide us innovative insights in DCs cancer immunology (Figure 4).

### 5.1 Female sex marks regulation of DCs

P4, which is present at higher levels in females, plays a key role in regulating DCs phenotype through interactions with both the PR and the glucocorticoid receptor (GR) signaling pathways. Studies have shown a correlation between elevated serum P4 levels and increased peripheral blood levels of IFN-α^+^ plasmacytoid dendritic cells (pDCs) 185. These pDCs inhibit the secretion of tumor necrosis factor (TNF) and IL-1β by DCs and downregulate their ability to activate T cells. This effect is likely due to the P4/PR and P4/GR signaling axes, which upregulate TLR7, Unc93b1, and TLR9 in female-derived DCs 186, 187.

Estrogen may exert similar effects on DCs by increasing the proportion of IFN-α^+^ DCs activated through TLR7 or TLR9 stimulation. This process involves the upregulation of Unc93b1 and IRF5 expression via estrogen receptor 1 (ESR1)-dependent pathways 39. Additionally, the ability of DCs to regulate the activation of other immune cells, as opposed to responding to immune cell activation, exhibits sex differences, primarily mediated by E2 and estrogen ERα signaling. E2/ERα signaling upregulates major histocompatibility complex (MHC) class II molecules and co-stimulatory molecules on DCs, enhancing their activation. This signaling also increases environmental GM-CSF and IRF levels, further boosting the antigen-presenting cells (APCs) efficacy 39.

In about 1% of severely ill male COVID-19 patients, defects in TLR7 have been identified, impairing the ability of pDCs to secrete IFN-I and provide protective immunity. This defect is linked to a variant of the *TLR7* gene located on the X chromosome 188. Additionally, increased TLR7 expression and heightened IFN signaling, influenced by X chromosome dosage, support the hypothesis that TLR7 escapes XCI, with estrogen further enhancing this effect 189, 190. As a result, female pDCs produce higher levels of IFN-α in response to TLR7 signaling compared to males, a difference that contributes to the pathogenesis of SLE and HIV-1 infection 191. Moreover, IL13RA1 and CYBB have also been shown to evade XCI in DCs, leading to enhanced DCs activation and phagocytosis 192. While the specific gender-differentiated effects within the TME remain under investigation, DCs appear to activate T-cell-dominated tumor immune responses more effectively in females, leading to improved tumor healing and prolonged survival.

### 5.2 Male sex marks regulation of DCs

In males, AR-mediated inhibitory effects on DCs primarily impact T cell maturation, differentiation, and pDCs function. Male infants, during early responses to viral infections when androgen levels are high, produce significantly lower levels of IFN-α from pDCs compared to females 193. Similarly, in adolescents (12-16 years of age) following mRNA vaccination, androgens delay pDCs activation and reduce IFN-α production, with this effect negatively correlating with free testosterone levels 194. Testosterone administration in transgender males also results in decreased IFN-I production by pDCs 195. In androgen-deprived male mice, surface expression of MHC-II and co-stimulatory molecules like CD80, CD83, CD86, CD40, and OX40L is downregulated on DCs in prostate cancer 196, 197. Direct exposure to dihydrotestosterone impairs activated T cell function in DCs 198. Furthermore, young male rats exhibit a higher proportion of immature DCs and a reduced ability to stimulate CD4^+^ T cell polarization compared to females 199. This observation is supported by studies in rhesus monkeys with leukemia, which show a lower number of myeloid DCs in males than in females 200. The absence of sex-specific differences in AR-deficient DCs suggests that androgen effects on DCs are AR-dependent 200.

Furthermore, sex differences significantly influence the phenotype of DCs across various diseases, with both estrogen and androgen receptors present in cancer tissues 201. However, despite these distinctions, significant sex differences have not been observed in the effectiveness of cancer therapies, such as DCs vaccine therapy 202. The application of sex-based strategies for tailored cancer treatment is still challenging at the level of DCs function and response.

## 6. Sex bias in Neutrophil tumor immunity

**Figure 5:**
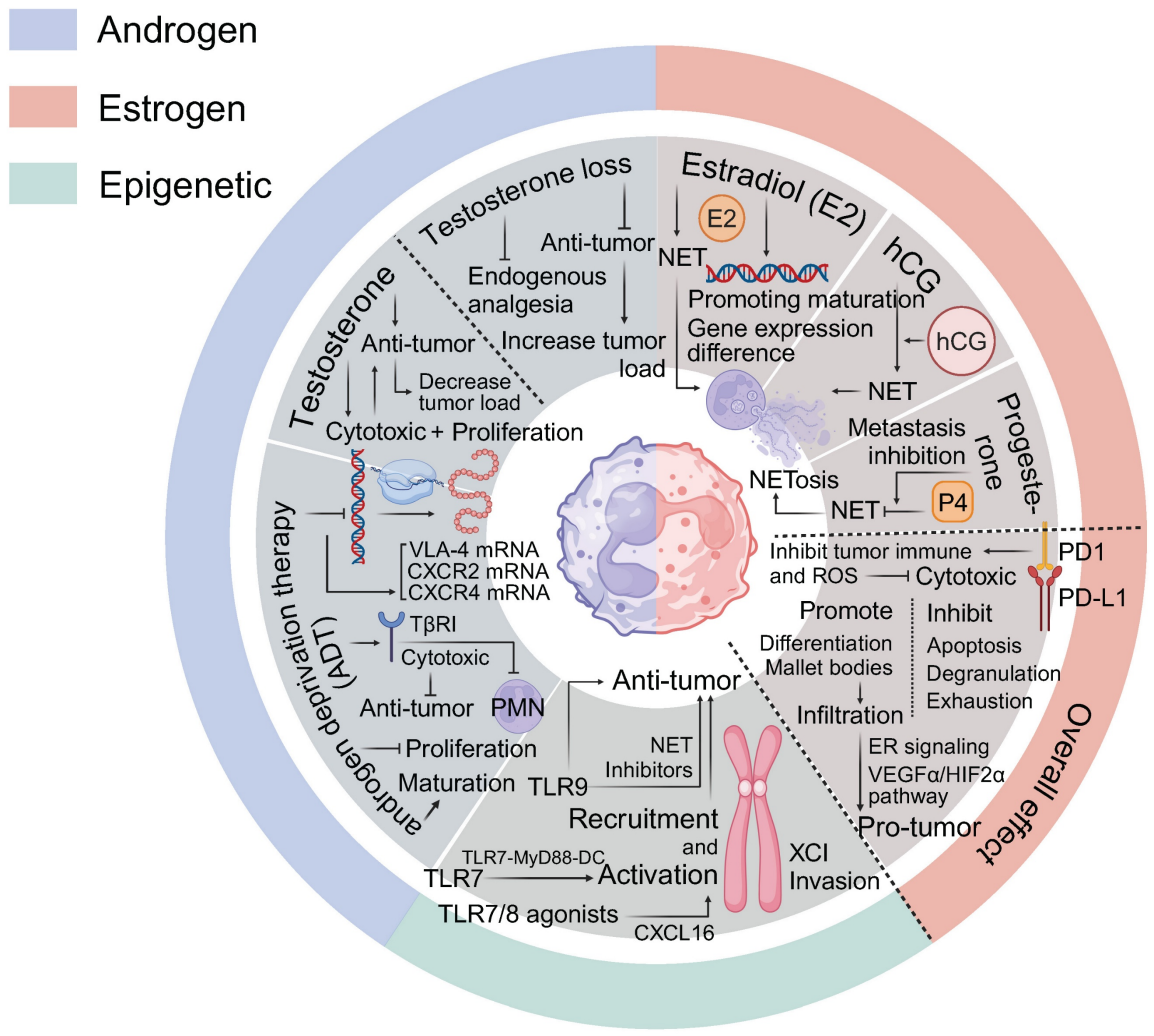
** Sex Factors in Regulating Neutrophils Tumor Immunity.** E2 plays a pivotal role in neutrophil function by promoting differentiation, inhibiting apoptosis, and enhancing NET and NETosis. In renal cell carcinoma, estrogen facilitates neutrophil infiltration into the TME via ER signaling and the VEGFα/HIF2α pathway, contributing to tumor progression. Additionally, estrogen inhibits neutrophil degranulation, which can suppress tumor immunoreactivity through the PD-1/PD-L1 pathway and affect oxygen-dependent intracellular killing mechanisms. Estrogen also depends neutrophils in female exhibiting a greater number of mallet bodies. HCG enhances NET formation, which can either promote tumor cell death or contribute to metastasis, depending on the context, highlighting its complex role in tumor biology. P4 acts as an antagonist to NETosis by inhibiting the transfer of NE from the cytoplasm to the nucleus, reducing NET formation and impacting neutrophil-mediated tumor immunity. Testosterone promotes the anti-tumor effect of neutrophil by enhancing its cytotoxic and proliferation, while loss of testosterone inhibits endogenous analgesia and increases tumor load. ADT impairs neutrophil function by inhibiting cytotoxic and proliferation through the increased expression of TβRI, thereby reducing the tumor immunoreactivity of neutrophils and PMN. Epigenetic modifications involving the X chromosome significantly influence neutrophil function. TLR7 and TLR9 genes, which evade XCI, are upregulated in neutrophils, enhancing their recruitment and activation through the TLR7-MyD88-DC pathway. The CXCL16 axis and TLR7/8 agonists further promote neutrophil recruitment, potentially leading to antitumor effects.

Neutrophils within tumor show notable sex-based heterogeneity, driven primarily by sex hormones, sex chromosomes and epigenetic factors. This variability impacts several key neutrophil functions, including activation, apoptosis, proliferative infiltration, surface checkpoint expression, and the formation of neutrophil extracellular traps (NETs) (Figure 5).

### 6.1 Estrogen regulation of Neutrophils

In hepatocellular carcinoma (HCC), neutrophils contribute to tumor progression through various mechanisms 203. For example, neutrophils in this context express PD-L1, which impairs tumor immune responses. Single-cell RNA sequencing(scRNA-seq) studies have demonstrated differential gene expression in E2-stimulated neutrophils, shedding light on how estrogen influences infiltrating macrophages and monocytes 204. Furthermore, mRNA sequencing has revealed that male neutrophils are generally more immature and metabolically active than their female counterparts 205. Estrogen plays a key role in promoting neutrophil differentiation and inhibiting apoptosis, leading to observed sex differences in neutrophil numbers and functionality 206, 207. Additionally, estrogen has been shown to inhibit neutrophil degranulation, which can suppress tumor immunoreactivity through the PD-1/PD-L1 pathway and impact oxygen-dependent intracellular killing mechanisms 208.

Both E2 and hCG have been shown to enhance NETs formation and NETosis. In contrast, P4 acts as an antagonist, inhibiting the efficient transfer of neutrophil elastase (NE) from the cytoplasm to the nucleus, thereby impeding NET formation 209. In the context of neutrophil exhaustion and the inhibition of NETosis, estrogen has been implicated in promoting aneurysm rupture. Conversely, estrogen-deficient mice demonstrate a decreased likelihood of aneurysm rupture 210. Estrogen also contributes to the sex-differentiated appearance of neutrophils, with females displaying a greater number of mallet bodies 211. Furthermore, estrogen facilitates neutrophil infiltration into the TME by modulating cellular communication through the ER pathway. This process plays a role in promoting renal cell carcinoma progression, with infiltrating neutrophils contributing to tumor advancement, particularly via ER signaling and the VEGFα/HIF2α pathway 212, 213.

### 6.2 Androgen regulation of Neutrophils

Androgens have been shown to negatively impact neutrophil function in the TME, but this perspective may not capture the full scope of their effects. GC illustrates a notable sex disparity, with incidence and mortality rates in males approximately twice as high as in females. Statistical analyses have revealed that tumor-associated neutrophil (TAN) density varies by GC type, with diffuse GC exhibiting the least TAN infiltration. Importantly, male patients demonstrate greater variability in TAN density within tumors compared to females, suggesting that TAN density may serve as a sex-specific prognostic indicator for GC 214, 215.

In melanoma models, male mice subjected to gonadal castration showed increased tumor burden, while testosterone treatment in female mice reduced tumor sizes. Furthermore, neutrophils from castrated male mice exhibited lower percentages and expressed reduced CXCR2 mRNA levels, alongside higher CXCR4 and VLA-4 mRNA levels, impairing neutrophil activation and immune responses 216. These findings underscore the influence of sex hormones on neutrophil numbers, gene expression, and cytotoxic functions 216. Additionally, in renal infections and inflammation, androgen exposure has been associated with impaired neutrophil maturation and function 217. Similarly, testosterone antagonists used in oral cancer models have been shown to mimic opioid effects, impacting neutrophil-mediated analgesia 218.

The observed reduction in neutrophil numbers and altered function following castration of male mice, compared to the myeloid-derived suppressor cell-like phenotype in prostate cancer patients undergoing ADT, emphasizes the complex role of androgens in modulating immune responses 216. These findings suggest that androgen-mediated changes in neutrophil function may contribute to tumor metastasis and progression 216. Further studies on ADT have revealed abnormalities in neutrophils from both ADT-treated patients and castrated male mice. ADT appears to impair neutrophil-mediated antitumor activity by inhibiting cytotoxic functions, likely through the increased expression of transforming growth factor β receptor I (TβRI) 219. This inhibition suppresses the tumor immunoreactivity of peripheral blood polymorphonuclear neutrophils (PMNs), potentially reducing their effectiveness in combating tumor growth 219.

### 6.3 Sex chromosome and epigenetic regulation of Neutrophils

The observed phenotypic differences in neutrophils can be attributed to epigenetic modifications, particularly those involving the X chromosome. Studies have shown that *TLR7* genes, by evading XCI, can upregulate their own expression, thereby enhancing neutrophil recruitment and activation through the TLR7-MyD88-DCs signaling pathway. Similarly, the CXCL16 axis and TLR7/8 agonists are effective in recruiting large numbers of neutrophils, potentially contributing to antitumor effects 220, 221. TLR9 agonists have also demonstrated substantial anticancer efficacy across various types of cancer, with TLR9 expression in neutrophils emerging as a crucial prognostic marker 222, 223. Notably, TLR9, which also evades XCI, interacts with NETs to inhibit Merlin phosphorylation, leading to resistance to ferroptosis in TNBC cells. Consequently, targeting NETs appears to be a promising therapeutic strategy for TNBC 224.

## 7. Sex bias in Macrophage tumor immunity

**Figure 6:**
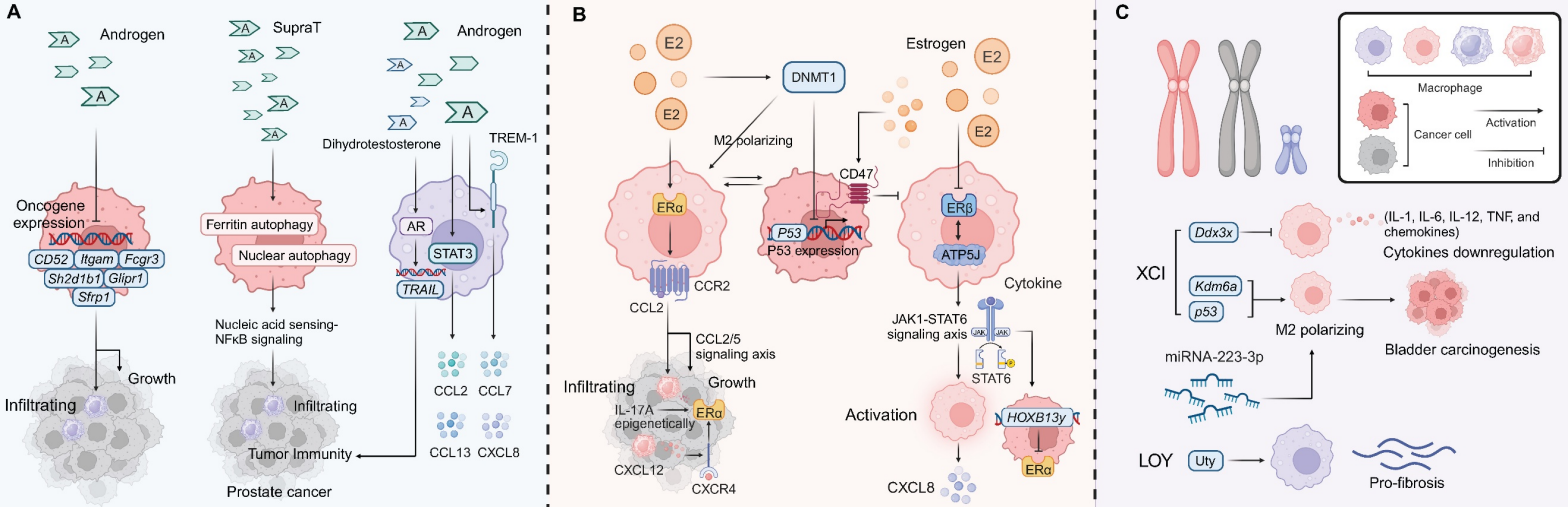
** Sex Factors in Regulating Macrophages Tumor Immunity.** (A) In melanoma, downregulation of AR signaling enhances macrophage infiltration, inhibits tumor progression, and may provide a basis for novel immunotherapeutic strategies. In prostate cancer, androgens such as testosterone and dihydrotestosterone activate STAT3 and upregulate genes like TREM-1, CCL2, CCL7, CCL13, and CXCL8, promoting metastasis. Additionally, in thyroid cancer, testosterone influences oncogene expression (e.g., CD52, Sh2d1b1, Fcgr3, Itgam, Glipr1, Sfrp1) and inhibits immune cell infiltration while SupraT can induce immune responses through ferritin and nuclear autophagy and thus through nucleic acid sensing-NFκB signaling in prostate cancer. AR signaling also promotes TRAIL gene transcription, enhancing macrophage cytotoxicity against prostate cancer cells. (B) In NSCLC, ERα activation drives the CCL2/CCR2 axis, promoting macrophage infiltration and cancer cell invasion, while upregulating CXCL12/CXCR4 signaling. Estrogen inhibits DNMT1, leading to decreased p53 expression and inducing M2 macrophage polarization. In endometrial cancer, IL-17A-mediated epigenetic mechanisms induce ERα expression in macrophages, which sensitizes cancer cells to estrogen and promotes tumor progression. Estrogen also upregulates CD47, inhibiting macrophage phagocytosis in ER+ tumor cells; blocking CD47 with anti-estrogens enhances macrophage phagocytosis. In ER+ breast cancer, estrogen stimulates macrophages through CCL2 and CCL5, contributing to tumor progression. (C) Female bone marrow-derived macrophages with deletion of the X-linked gene Ddx3x exhibit impaired innate immune responses, including reduced cytokine production (IL-1, IL-6, IL-12, TNF) and a diminished ability to restrict bacterial proliferation. The Kdm6a and p53 gene, and miRNA-223-3p, which promotes M2 macrophage polarization, contribute to bladder carcinogenesis. The LOY disrupts the Y-linked gene Uty, promoting pro-fibrotic macrophage phenotypes.

Androgens, estrogens, Sex chromosomes and epigenetic factors have a profound impact on macrophage function in tumor, influencing both cancer progression and immune responses through various mechanisms (Figure 6).

### 7.1 Androgen regulation of macrophages

AR signaling plays a key role in regulating macrophage infiltration, cytotoxicity, and immune responses. In melanoma, downregulation of AR signaling has been shown to increase macrophage infiltration, inhibit tumor progression, and offer potential avenues for immunotherapy 225. In contrast, AR signaling can enhance macrophage-driven promotion of breast cancer progression 226. In prostate cancer, androgen stimulation mediates metastasis through STAT3 activation and the upregulation of inflammatory cytokines such as TREM-1, CCL2, CCL7, CCL13, and CXCL8 227, 228. Additionally, in thyroid cancer, Thrb^PV/PV^ mice, testosterone influences oncogene expression, including *CD52*, *Sh2d1b1*, *Fcgr3*, *Itgam*, *Glipr1*, and *Sfrp1*, and inhibits macrophage and other immune cell infiltration 229. In colorectal and prostate cancer, males are more biased to show more anti-tumor M1 polarization while more M2 phenotype is demonstrated in females 24, 228. Supraphysiologic levels of testosterone (SupraT) can induce immune responses by activating ferritin and nuclear autophagy, subsequently triggering nucleic acid sensing-NFκB signaling in prostate cancer 230. Moreover, dihydrotestosterone promotes the transcription of *TRAIL* genes via AR signaling, enhancing macrophage cytotoxicity against prostate cancer cells 231. A similar effect and sex bias have also been observed in immune checkpoint blockade (ICB) therapy for melanoma, where male patients derive greater benefit from ICB treatment compared to females, primarily influenced by the functionality of intratumoral macrophages 232.

### 7.2 Estrogen regulation of macrophages

Estrogen regulates macrophage activity and tumor progression through interactions with a wide range of cytokines and signaling pathways. Estrogen upregulates CD47 in ER^+^ tumor cells, inhibiting macrophage-mediated phagocytosis 233, 234. Inhibition of CD47 with anti-estrogens has been shown to enhance macrophage phagocytosis, suggesting a potential therapeutic strategy 234. Additionally, estrogen promotes tumorigenesis in normal breast tissue by increasing the number of CD206^+^ macrophages, while simultaneously reducing the presence of dendritic cells and T cells. Conversely, oophorectomy, which reduces estrogen levels, leads to an increase in M1 macrophages and has been associated with slowed progression in ovarian cancer 235, 236.

ER signaling enhances tumor immunity by reducing immune checkpoint resistance, with tumor-associated macrophages (TAMs) and myeloid-derived suppressor cells (MDSCs) playing a central role in this process. In endometrial cancer, uterine-infiltrating CD68^+^CD163^+^ macrophages induce ERα expression through IL-17A-mediated epigenetic mechanisms, sensitizing cancer cells to E2 and accelerating tumor progression 237. In breast cancer, estrogen stimulates macrophage activity via CCL2 and CCL5, while in endometrial cancer, macrophage-induced ERα expression enhances tumor progression 238. In HCC, estrogen represses tumor growth by inhibiting the interaction between ERβ and ATP5J, blocking the JAK1-STAT6 axis, and inhibiting TAM alternative activation, which in turn secretes CXCL8. This leads to the loss of ERα in endometrial carcinoma (EC) through HOXB13y expression, contributing to a poor prognosis 239, 240. In NSCLC, ERα activation triggers the CCL2/CCR2 axis, promoting macrophage infiltration and facilitating cancer cell invasion, while also upregulating the CXCL12/CXCR4 signaling pathway 241. Furthermore, a series of reciprocal positive feedback loops between macrophages and cancer cells have been observed in both ER^+^ breast cancer and TNBC patients 242-244.

Estrogen-induced inhibition of DNMT1 results in decreased p53 expression, which in turn promotes M2 macrophage polarization, contributing to a poor prognosis in lung cancer 245. Estrogen/ERα-induced infiltration of TAMs and M2 polarization have also been shown to promote cancer progression 246, which promotes CD8^+^ T cell dysfunction and exhaustion and ICB resistance 232. Additionally, a study highlighted the potential therapeutic utility of tamoxifen in suppressing brain metastasis of hormone receptor-deficient breast cancer by blocking M2 polarization of microglia and enhancing their anti-tumor phagocytic activity 247. Exogenous E2 treatment reduces the pro-inflammation phenotype in macrophage cells and accelerates resolution of inflammation through IL-10 activation 64. Interestingly, 2-methoxyestradiol (2ME2), a metabolite of 17β-estradiol, has been shown to suppress M2 polarization and the pro-tumoral functions of macrophages in breast cancer by inhibiting STAT3. This suggests that further investigation is still needed to explore the potential estrogen-related pathways in macrophage polarization 248.

### 7.3 Sex chromosome and epigenetic regulation of macrophages

In addition to sex steroid hormone-mediated differences in macrophage immunity, sex chromosome and epigenetic factors also play a significant role in the observed sex variability in macrophage immune responses. For example, female bone marrow-derived macrophages with a deletion of the X-linked gene *Ddx3x* exhibit impaired ability to restrict the proliferation of *Listeria monocytogenes* and show a marked reduction in the expression of key cytokines, such as IL-1, IL-6, IL-12, TNF, and various chemokines 50. This deficiency in *Ddx3x* disrupts their functional response in innate immunity. Moreover, the *Kdm6a* gene, which has oncogenic properties when overexpressed in NK cells, has been found to be deficient in macrophages. This deficiency promotes M2 macrophage polarization and contributes to bladder carcinogenesis, particularly in the context of p53 deficiency 249. On the other hand, bladder cancer cells can be effectively inhibited by miRNA-223-3p, an X-chromosome-linked miRNA that regulates macrophage polarization. miRNA-223-3p reduces M1 levels, thereby diminishing the inflammatory response. This results in a relative disadvantage for females in terms of innate immune responses, due to the escape mechanisms of XCI that allow for the upregulation of immune-modulatory genes like miRNA-223-3p 250. Additionally, the loss of Y chromosome chimerism, which is often associated with changes in the X chromosome, leads to the disruption of the Y-linked gene *Uty*. This disruption has been found to promote pro-fibrotic macrophage phenotypes, which in turn contribute to the development and progression of sarcoma 251, 252.

## 8. Sex bias in other immune cells and pathways

Despite limited research on sex disparities in immune cells like MDSCs, innate lymphoid cells (ILCs), mast cells, eosinophils, and basophils within tumor immunology, sex-dependent biases are evident.

MDSCs associated with chronic inflammation include both mononuclear MDSCs (mMDSCs) and granulocytic MDSCs (gMDSCs). These cells primarily inhibit CTLs and NK cells within the TME, thereby promoting tumor angiogenesis, invasion, and metastasis 253, 254. In female patients, a higher level of peripheral gMDSCs is linked to poor prognosis, whereas in males, tumor-infiltrating mMDSCs are more prominent 255. E2 facilitates the accumulation of MDSCs in the bloodstream by stimulating the secretion of TNF-α in vivo 254. Additionally, E2-treated MDSCs, particularly mMDSCs, interact with T cells in melanoma, leading to T cell exhaustion and reduced cytotoxic function 232. In contrast, androgens downregulate MDSCs, thereby diminishing myeloid cell-mediated immune suppression and inhibiting tumor progression 256. However, following ADT, MDSC levels increase, which poses a persistent challenge and contributes to the progression of many patients to castration-resistant prostate cancer (CRPC) 256, 257.

Estrogen enhances ILCs tissue infiltration, infection resistance, and cytokine secretion, while androgens inhibit these functions via ER/AR pathways 39, 258-264. In tissue immunity, the androgen-ILC2-DCs axis negatively regulates sex differences in skin immunity, with androgens suppressing ILC2s 265. Estrogen and progesterone pathways in mast cells promote IgE-induced degranulation and leukotriene production, but the androgen effect varies depending on cell sources and subtypes 266. Eosinophils are influenced by estrogen binding to membrane ERα, activating GPR-1, which triggers immune responses such as proliferation and anti-tumor effects 267-270. In studies on pancreatic cancer development, eosinophils and ILCs are also crucial targets of sex-based regulation in the cancer-microbiome interactions 271.

Further research is required to identify the sex-dependent epigenetic and chromosomal factors that contribute to the dimorphism of MDSCs and the specific role and mechanisms of sex factors in the sex bias of ILCs, eosinophils, and basophils remains unclear, warranting further investigation. Beyond the estrogen and androgen signaling pathways previously discussed, other sex-differentiated molecules, such as FSH, LH, and CRH, also influence immune responses (Table 1). However, the impact of most molecules on immunity in tumor immunity still needs to be explored.

## 9. Discussion

This review explores the roles of sex chromosomes, sex hormone levels, and sex hormone receptor expression in modulating immune cell behavior, which varies significantly between sexes and influences treatment responses and adverse effects. In general, females exhibit stronger innate and adaptive immune responses than males 272, which may partially explain the higher mortality rates and shorter survival times observed in male patients 2. However, males often show more favorable responses to immune checkpoint blockade therapy, while females tend to experience more severe adverse events 12. Additionally, females typically present with more aggressive, advanced, and refractory disease courses, characterized by higher recurrence rates compared to males. This disparity may be linked to the stimulatory effects of sex hormones on cell proliferation, drug resistance, and the inhibition of apoptosis in cancer cells 77.

Significant sex differences also influence the response to radiotherapy, chemotherapy, and emerging targeted therapies and immunotherapies 273, 274. Gender-related oxidative stress, regulated by sex hormones, impacts redox state proteins and mitochondrial function, potentially altering the efficacy and toxicity of cancer treatments. This mechanism may contribute to the observed greater resistance to cancer in young females compared to males 275. Moreover, chemotherapy-induced cardiotoxicity is more prevalent in females, while tyrosine kinase inhibitors are more likely to cause vascular embolism in female patients, potentially due to reduced nitric oxide (NO) synthesis and endothelial dysfunction 276. Additionally, radiotherapy-induced tissue hypomethylation is more pronounced in male patients 275. These findings highlight the importance of considering sex as a critical factor when evaluating the risks and benefits of cancer therapies.

Sex steroid levels fluctuate significantly across different life stages, including puberty, menopause, and the reproductive phase, leading to considerable variations in hormone concentrations and signaling through sex steroid receptors 64. Menstrual and reproductive factors significantly influence cancer risk. Nulliparity, or never having given birth, is a well-established risk factor for several cancers, including luminal breast cancer, ovarian cancer, and endometrial cancer 277-280. However, the relationship between parity and cancer risk is more complex, especially in Black women, where high parity has been linked to a higher risk of triple-negative breast cancer (TNBC) 281. While the disparity between nulliparity and ever-parity was traditionally thought to stem from differences in ovulatory cycles, recent studies suggest that parous women may have an increased frequency of mutations in breast stromal cells 282, 283. The timing of menopause also plays a crucial role in cancer risk. Later menopause is associated with a higher incidence of hormone-related cancers, such as breast, endometrial, ovarian, and colon cancers, while early menopause has been linked to an increased risk of lung cancer 284-286. For pregnant patients with breast cancer, the 5-year disease-free survival (DFS) rate increases from 65% to 71% if they are not pregnant, and the 5-year OS rate rises from 78% to 81% 287. Similarly, pregnant women with melanoma tend to have lower survival rates compared to their non-pregnant counterparts 288. Beyond gynecological cancers, the incidence of lung and colon cancers also rises in perimenopausal female, with poorer prognosis compared to postmenopausal group 289, 290. In serous ovarian cancer ascites, the premenopausal group shows an increased proportion of T cells, while tumor immunity in the postmenopausal group is more associated with the IL-17 pathway 291. These findings highlight the alteration of immune cell functions in response to varying estrogens over female life course 64.

Hormone replacement therapy (HRT) is another significant factor. Although HRT increases the risk of breast and ovarian cancers, it has been shown to reduce the risk of esophageal, colon, and gastric cancers 292-296. Notably, HRT regimens containing both estrogen and progesterone are linked to a higher risk of breast cancer compared to estrogen-only therapies. Additionally, the cancer risk associated with HRT is more pronounced in estrogen receptor-positive tumors than in estrogen receptor-negative ones. Importantly, the increased risk of breast cancer diminishes within two years after discontinuing HRT 297.

Sex chromosomes play a crucial role in regulating tumor immune responses. The X chromosome contains approximately 50 genes involved in immune functions, including those responsible for immune cell identification (*FOXP3*), cellular activation and intracellular signaling (*CD40LG*, *TLR7*, *IRAK1*, *IL13RA1/2*, *NEMO*, *TASL*, *IL9R*), immune cell differentiation and proliferation (*IL2RG*, *BTK*), and cellular metabolism (*OGT*) 272. Furthermore, autosomal genes regulating immune pathways can also be influenced by X chromosome inactivation (e.g., *EIF1AX* and *KDM6A*) 298. The long non-coding RNA X-inactivation-specific transcript (XIST), which is regulated by the oncogene *P53*, has complex effects on cancer, with both protective and promotive roles 299, 300. Increased cancer risk in females is partly attributed to the loss or dysfunction of tumor suppressor genes that escape XCI, such as *ATRX*, *CNKSR2*, *DDX3X*, *KDM5C*, *KDM6A*, *MAGEC3*, and other X-inactivating tumor suppressors (EXITS) 20.

Furthermore, developmental XCI results in X-chimerism, enabling the preferential expression of specific mosaic subpopulations of the X chromosome in female cells. This process may enhance the immune system's ability to defend against cancer 301, 302. In contrast, males exhibit an increased risk of cancer due to the LOY and the extreme downregulation of Y-linked genes (EDY), both of which serve as valuable biomarkers for cancer prediction 303. Incomplete inactivation of the X and Y chromosomes, along with the overexpression of sex chromosome-linked genes in various immune cells—such as T cells, DCs, and NK cells—can contribute to cellular exhaustion and diminished tumor immunoreactivity 21.

Sex hormones and their receptors play a critical role in orchestrating molecular and cellular processes that influence cancer risk and progression. For example, estrogen signaling has been shown to reduce circulating IL-6 levels and inhibit the secretion of inflammatory cytokines by macrophages and neutrophils, thereby lowering the risk of lung and liver cancer in females 304, 305. In the adaptive immune system, estrogen can upregulate several genes, such as *IFN-γ*, *IFI6*, *CX3CL1*, *CX3CL2*, *IL-1*, *IL-5*, and *IL-16* in T cells, which are associated with enhanced inflammatory and cytotoxic T-cell responses 306. Estrogen also directly regulates other immune cell types, including B cells, dendritic cells, and pDCs, all of which are promising targets for immunotherapy 307, 308. However, estrogen's role in cancer progression can be dual. It can promote cancer by increasing intracellular PD-1 expression and decreasing p53 expression 309, 310.

Regarding epigenetic effects, androgens have been observed to reduce DNA methylation in embryonic neural stem cells and liver cells in males, creating epigenetic patterns similar to those seen in cancer cells 311, 312. Additionally, both estrogen and androgens can contribute to tumorigenesis through the mTOR signaling pathway 313. Sex hormones also influence cancer progression by promoting endothelial cell proliferation and migration. This occurs through the regulation of angiogenic genes, the stimulation of endothelial nitric oxide synthase (eNOS) production, and the activation of mesenchymal stromal cells to secrete factors like VEGF. These effects are mediated by ERs, PRs, and ARs 79, 314-316.

Numerous drugs targeting sex hormone pathways are approved or in development for cancer treatment, including immunotherapies, chemotherapies, and targeted therapies. These include AR antagonists (biclautamide, nilutamide, darolutamide, apalutamide and enzalutamide), LHRH/GnRH agonists (leuprolide, goserelin and buserelin), LHRH/GnRH antagonists (degarelix, relugolix and abarelix), selective ER modulators and downregulators (tamoxifen, raloxifene and fulvestrant), GPER agonists (LNS8801d), aromatase inhibitors (exemestane, letrozole and anastrozole) 12, 317. Several clinical trials exploring sex hormone-based therapies in non-reproductive organ-derived cancers have been conducted, with the potential mechanisms of modulating anti-cancer immune responses still under investigation (Table 2).

Additionally, therapies such as adoptive T cell therapy, immune checkpoint inhibitors, angiogenesis and tyrosine kinase inhibitors, EZH1/2 and BET inhibitors (targeting sex-specific epigenetic and X chromosome inactivation) show sex dimorphism in anti-cancer treatment. Proportion of the treatments are more effective when combined with sex hormone-targeting drugs 317, 318. Recently, novel cancer drugs with sex bias like telomerase reverse transcriptase (TERT) modulators are emerging, while drugs targeting sex-related genetic factors like telomerase inhibitors still require further investigation 319. Furthermore, a research revealed treatment biases, highlighting the urgent need for comprehensive sex-based analyses to uncover more clinically significant findings and support the development of personalized, sex-specific cancer treatments 320.

In addition to the previously discussed sex-biased cellular and molecular mechanisms, further investigation is needed to explore sex differences in cancer from the following perspectives: (i) sex-biased cellular senescence in immune cells, as this may play a role in the observed differences in immune-related diseases between sexes 321. (ii) The impact of sex-specific microbiota on tumor immune responses, since commensal microbiota can produce sex steroids, like androgens, that influence the immune landscape and disease outcomes 322. (iii) Sex-biased metabolism, which may lead to differences in immune cell function 323. Notably, significant sex differences in metabolism—such as glycolysis, fatty acid, and bile acid metabolism—have been observed across 13 non-reproductive cancers in the TCGA dataset 323. (iv) Sex-specific variations in the response to persistent DNA damage signaling, which are critical in managing the tissue-level effects of DNA damage.

In recent research, sex-based differences in the cancer microbiota contributing to cancer progression (colorectal cancer, especially) has been found 324-327, beyond the autoimmunity 328, inflammation 329, or neural and respiratory diseases 330, 331. In colorectal cancer, the molecular mechanisms of sex-based disparities are focused on sex hormone-gut microbiome axis 324. Sex-based sexual dimorphism extends to microbe-associated molecular patterns (MAMPs), where estrogens promote greater gut flora diversity, while androgens tend to reduce microbial diversity. This differential impact on the microbiome is associated with cancer development 332. However, to advance preclinical research and accelerate clinical treatment, challenges remain in fully understanding the role and molecular mechanisms of microbiome-related sexual dimorphisms in cancer. Further investigation is needed to address these gaps.

Understanding sex-biased factors in tumor immunity is crucial for developing sex-specific therapies. In 2016, the U.S. National Institutes of Health (NIH) introduced a policy requiring investigator-initiated grants to consider sex as a biological variable in medical research 322. Historically, over 60% of immunology studies using animal models did not report the sex of the subjects as of 2009 333. However, by 2014, half of the published studies included both sex and age as experimental variables 334. The significant sex differences observed across all levels of biological organization underscore the fact that findings in males cannot be universally applied to females, and vice versa 333. Despite this, women remain significantly underrepresented in immunotherapy clinical trials 335, potentially due to concerns that cyclical hormonal changes may influence clinical outcomes 336. Therefore, further research is needed to understand sex hormone-independent cancer mechanisms. Additionally, sex balance should be carefully considered in clinical trial design before gender-specific targeted therapies are implemented in clinical practice.

## Figures and Tables

**Table 1:** Other sex biased molecules regulation of immunity.

Molecules	Dominant sex	Regulation of female/male immunity	Refs
Activin/Inhibin	Female	Impairing tumor immunity by affecting CD8+ T cells.	337, 338
Bile Acids	Male	Upregulating FOXP3+ Tregs and interfering CRC prognosis.	339
Catecholamines	Male	Activating T cells, inflammation and autoimmunity.	340
Collagen	Female	Impairing T cells cytotoxicity, stimulating its migration and M2 polarization.	341
DHEA	Male	Stimulating Th1 response and Tregs, impairing Th17 response.	342
FSH	Female	FSH downregulation impairs anti-tumor immunity in males.	343
hCG	Female	Stimulating lymphocyte secretion of IL-10 while downregulating TNF.	344
NP	Female	Inhibiting inflammasome activation in immune cells.	345, 346
OT	Female	Activating T cells while inhibiting the infiltration of neutrophils and macrophages, and suppressing the expression of pro-inflammatory cytokines such as TNF-α and IL-1β.	347
PRL	Female	Promoting T cells, B cells, NK cells, DCs, etc. activation and response.	348
VP	Male	Stimulating neutrophils and monocytes, thereby promoting inflammation.	349, 350

CD: cluster of differentiation; CRC: colorectal cancer; FOXP3: forkhead box P3; M2: M2 macrophages; DHEA: dehydroepiandrosterone; Th: helper T cell; FSH: follicle stimulating hormone; hCG: human chorionic gonadotropin; TNF: tumor necrosis factor; NP: natriuretic peptides; OT: oxytocin; IL: interleukin; PRL: prolactin; NK cells: natural killer cells; DCs: dendritic cells; VP: vasopressin.

**Table 2:** Registered clinical trials of sex hormone-based therapy conducted in non-reproductive organ derived cancers.

NCT identifier	Trial titles	Phase	Population description	Status	Cancer type	Publication
NCT00002595	Toremifene in Treating Patients with Desmoid Tumors	Phase II	n=72 (Estimated); Drug (Target): Toremifene (SERM); Procedure: conventional surgery	Completed	Desmoid Tumor	NA
NCT02353429	Toremifene in Desmoid Tumor: Prospective Clinical Trial and Identification of Potential Molecular Targets	Phase II	n=25 (Estimated); Drug (Target): Toremifene (SERM)	Unknown status		NA
NCT00068419	Sulindac and Tamoxifen in Treating Patients with Desmoid Tumor	Phase II	n=70; Drug (Target): Tamoxifen citrate (SERM)/Sulindac (COX-1/COX-2 inhibitor); Other: laboratory biomarker analysis	Completed		NA
NCT01642186	Study of (1) Everolimus, (2) Estrogen Deprivation Therapy (EDT) With Leuprolide + Letrozole and (3) Everolimus + EDT in Patients with Unresectable Fibrolamellar Hepatocellular Carcinoma (FLL-HCC)	Phase II	n=28; Drug (Target): Everolimus (mTOR inhibitor)/Letrozole (Estrogen inhibitor)/Leuprolide (LHRH/GnRH agonist)	Completed	Fibrolamellar Liver Cancer	351
NCT01402648	Estrogen Receptor Beta Agonists (Eviendep) and Polyp Recurrence	Phase I/II	n=60; Dietary Supplement (Target): Eviendep (CM&D Pharma Limited, UK) (ERβ agonist)/Maltodextrins (Placebo)	Completed	Gastrointestinal Neoplasm	NA
NCT02089386	Tamoxifen to Treat Barrett's Metaplasia	Early Phase I	n=7; Drug (Target): Tamoxifen (SERM)	Terminated		NA
NCT02513849	Tamoxifen in Patients with Oesophageal Cancer	Phase I	n=20 (Estimated); Drug (Target): Tamoxifen (SERM)	Unknown status		NA
NCT00024336	Radiation Therapy and Tamoxifen in Treating Children with Newly Diagnosed Brain Stem Glioma	Phase II	NA; Drug (Target): Tamoxifen citrate (SERM); Radiation: radiation therapy	Unknown status	Glioblastoma	NA
NCT04765098	Tamoxifen Versus Etoposide After First Recurrence in GBM Patients	Phase II	n=60 (Estimated); Drug (Target): Etoposide (Topoisomerase II inhibitor)/Tamoxifen (SERM)	Recruiting		NA
NCT06501911	A Study of Bicalutamide with Brain Re-irradiation to Treat Recurrent/Progressive High Grade Glioma	Phase I	n=30 (Estimated); Drug (Target): Bicalutamide (AR inhibitor); Radiation: Intensity-modulated radiation therapy (IMRT)	Not yet recruiting		NA
NCT00004436	Randomized Study of Hormonal Regulation of Infantile Hemangioma	n/a	n=30; Drug (Target): Leuprolide (LHRH/GnRH agonist)/Prednisone (Glucocorticoid)	Completed	Hemangioma	NA
NCT02528643	A Study to Assess the Efficacy and Safety of Enzalutamide in Subjects with Advanced Hepatocellular Carcinoma	Phase II	n=165; Drug (Target): Enzalutamide (AR inhibitor)/Placebo	Completed	Lung Cancer	352
NCT00003424	Tamoxifen in Treating Patients with Primary Liver Cancer	Phase III	n=300 (Estimated); Drug (Target): Tamoxifen citrate (SERM)	Completed		NA
NCT02642939	Study of Oral Mifepristone as Salvage Therapy in Patients with Advanced or Metastatic Non-Small Cell Lung Cancer	Phase II	n=3; Drug (Target): Mifepristone (GR-II receptor/PR/AR inhibitor)	Terminated		NA
NCT06512207	A Study on the Efficacy of Androgen Deprivation Therapy Combined with Anti-PD-1 Therapy in Advanced Lung Cancer	n/a	n=80 (Estimated); Drug (Target): Leuprorelin acetate (Androgen inhibitor)/Sintilimab (PD-1 inhibitor)	Recruiting		NA
NCT01556191	Lung Cancer in Women Treated with Anti-oestrogens anD Inhibitors of EGFR (LADIE)	Phase II	n=379; Drug (Target): Gefitinib (EGFR tyrosine kinase inhibitor)/Fulvestrant (ER inhibitor)/Erlotinib (EGFR tyrosine kinase inhibitor)	Completed		353
NCT00592007	Study Evaluating the Addition of Fulvestrant to Erlotinib in Stage IIIB/IV Non-Small Cell Lung Cancer	Phase II	n=7; Drug (Target): Fulvestrant (ER inhibitor)/Erlotinib (EGFR tyrosine kinase inhibitor)	Terminated		NA
NCT02666105	Exemestane in Post-Menopausal Women With NSCLC	Phase II	n=6; Drug (Target): Exemestane (Estrogen inhibitor)	Completed		NA
NCT02489123	Enzalutamide in Treating Patients with Relapsed or Refractory Mantle Cell Lymphoma	Phase II	n=8; Drug (Target): Enzalutamide (AR inhibitor); Other: Laboratory Biomarker Analysis	Terminated	Lymphoma	NA
Debio 8200-IMM-101	A phase I study on the safety and efficacy of triptorelin in combination with nivolumab in men with advanced melanoma resistant to prior anti-PD-1/PD-L1 therapy	Phase I	n=14; Drug (Target): Triptorelin (GnRH agonist)/Nivolumab (PD-1 inhibitor)/Bicalutamide (AR inhibitor)	Completed	Melanoma	354
NCT00254397	Melanoma Vaccine with Peptides and Leuprolide	Phase II	n=98; Drug (Target): Leuprolide (LHRH/GnRH agonist); Biological: GP100: 209-217(210M) Peptide/MAGE-3 Peptide	Completed		NA
NCT06320990	Chemoprevention With Tamoxifen in Pre-Invasive Pancreas Mucinous Cystic Neoplasms Not Undergoing Immediate Resection (MCN_Tam)	Phase I	n=15 (Estimated); Drug (Target): Tamoxifen (SERM)	Not yet recruiting	Pancreatic Neoplasm	NA
NCT06222593	Study to Evaluate the Safety and Efficacy of Bicalutamide in Combination with Sunitinib in Patients with TKIs-resistant RCC	Phase I/II	n=28 (Estimated); Drug (Target): Bicalutamide (Androgen inhibitor)/Sunitinib (CYP3A4 inhibitor)	Not yet recruiting	Renal Cell Cancer	NA
NCT02885649	Enzalutamide Before Surgery in Treating Patients with Kidney Cancer	Early Phase I	n=3; Drug (Target): Enzalutamide (AR inhibitor); Other: Laboratory Biomarker Analysis; Procedure: Nephrectomy	Terminated		NA
NCT03169764	QUILT-3.047: NANT Head and Neck Squamous Cell Carcinoma (HNSCC) Vaccine: Combination Immunotherapy in Subjects with HNSCC Who Have Progressed on or After Chemotherapy and PD-1/PD-L1 Therapy	Phase I/II	n=0; Drug (Target): Fulvestrant (ER inhibitor)	Withdrawn	Squamous Cell Carcinoma	NA
NCT02605863	Enzalutamide for Bladder Cancer Chemoprevention	Phase II	n=1; Drug (Target): Enzalutamide (AR inhibitor)	Terminated	Urethral bladder cancer	NA
NCT02197897	Evaluation the Treatment of Tamoxifen of Low/Intermediate Risk Bladder Tumors (BCTamoxifen)	Phase II	n=15; Drug (Target): Tamoxifen citrate (SERM)	Completed		NA
NCT06018116	A Canadian Trial of Bicalutamide in Patients Receiving Maintenance Avelumab for Metastatic Urothelial Cancer. (CANUCK-01)	Phase II	n=0; Drug (Target): Bicalutamide (AR inhibitor)/Placebo	Withdrawn		NA
NCT00710970	Tamoxifen for Progressive Transitional Cell Carcinoma Following Previous Chemotherapy Treatment	Phase II	n=28; Drug (Target): Tamoxifen (SERM)	Completed		355, 356
NCT03197571	QUILT-3.048: NANT Urothelial Cancer Vaccine: Combination Immunotherapy in Subjects with Urothelial Cancer Who Have Progressed on or After Chemotherapy and PD-1/PD-L1 Therapy	Phase I/II	n=0; Drug (Target): Fulvestrant (ER inhibitor)	Withdrawn		NA

AR: androgen receptor; COX: cyclooxygenase; EGFR: epidermal growth factor receptor; ER: estrogen receptor; GBM: glioblastoma multiforme; GnRH: gonadotropin-releasing hormone; LHRH: luteinizing hormone-releasing hormone; mTOR: mammalian target of Rapamycin; NSCLC: non-small cell lung cancer; PD-1: programmed death receptor 1; PD-L1: programmed death ligand 1; PR: progesterone receptor; RCC: renal cell carcinoma; SERM: selective estrogen receptor modulator.

## Abbreviations

2ME2: 2-Methoxyestradiol
ADT: androgen deprivation therapy
APCs: antigen-presenting cells
AR: androgen receptor
AREs: androgen response elements
ARKO: androgen receptor knockout
BAFF: B-cell activating factor
BMDCs: bone marrow-derived cells
Bregs: regulatory B cells
CAR-T: antigen receptor T cell
CLL: chronic lymphocytic leukemia
CO19ORS: COVID-19-specific immune responses
CpG: cytosine-phosphate-guanine dinucleotide motif
CRPC: castration-resistant prostate cancer
cTECs: cortical thymic epithelial cells
CTLs: cytotoxic T lymphocytes
DCs: dendritic cells
DFS: disease-free survival
DHT: dihydrotestosterone
DLBCL: diffuse large B-cell lymphoma
Dll4: Delta-like 4
E2: 17β-estradiol
EC: endometrial carcinoma
EDY: extreme downregulation of Y-linked genes
EFF: effector T cells
eNOS: endothelial nitric oxide synthase
ERK: extracellular signal-regulated kinase
ER: estrogen receptor
ERα: estrogen receptor alpha
ERβ: estrogen receptor beta
ERE: estrogen response element
ESR1: estrogen receptor 1
ESR2: estrogen receptor 2
GC: Gastric cancer
gMDSCs: granulocytic MDSCs
GPR: G protein-coupled receptor
GR: glucocorticoid receptor
HCC: hepatocellular carcinoma
hCG: human chorionic gonadotropin
HRT: hormone replacement therapy
iAR: intracellular androgen receptors
JNK: c-Jun N-terminal kinase
ICB: immune checkpoint blockade
ICIs: immune checkpoint inhibitors
ICT: immune checkpoint inhibitor therapy
IFN-I: type I interferon
IFN-γ: interferon gamma
ILCs: innate lymphoid cells
KO: knockout
KS: klinefelter syndrome
LOY: Y chromosome mosaic loss
MAMPs: microbe-associated molecular patterns
MAPK: mitogen-activated protein kinase
mAR: membrane androgen receptors
MDSCs: myeloid-derived suppressor cells
MHC: major histocompatibility complex
mMDSCs: mononuclear MDSCs
NE: neutrophil elastase
NETs: neutrophil extracellular traps
NIH: national Institutes of Health
NSCLC: non-small cell lung cancer
OS: overall survival
P4: progesterone
PAAD: pancreatic adenocarcinoma
PD-1: programmed death-1
pDC: plasmacytoid dendritic cell
PEX: progenitor exhausted T cells
PFS: progression-free survival
PI3K: phosphatidylinositol 3 kinase
PIBF: progesterone-induced blocking factor
PKC: protein kinase C
PMNs: polymorphonuclear neutrophils
PPP: pentose phosphate pathway
PR: progesterone receptor
PRL: prolactin
PTC: papillary thyroid carcinoma
RBP: regulatory binding protein
scRNA-seq: Single-cell RNA sequencing
SERMs: selective estrogen receptor modulators
SLE: systemic lupus erythematosus
SupraT: supraphysiologic levels of testosterone
SupraT: supraphysiologic levels of testosterone
T-ALL: T-cell acute lymphoblastic leukemia
TAMs: tumor-associated macrophages
TAN: tumor-associated neutrophil
TCF: T cell factor
TCR: T cell receptor
TEX: terminally exhausted T cells
TERT: telomerase reverse transcriptase
Tfm: testicular feminization
Th: T helper cell
TIL-Bs: Tumor-infiltrating B lymphocytes
TKT: transketolase
TLS: tertiary lymphoid structures
TME: tumor microenvironment
TNBC: triple-negative breast cancer
TNF: tumor necrosis factor
Treg: regulatory T cell
TβRI: transforming growth factor β receptor I
UTX: ubiquitously transcribed tetratricopeptide repeat on chromosome X
VAT: visceral adipose tissue
XCI: X chromosome inactivation
XIST: RNA X-inactivation-specific transcript
